# Camelot: a computer-automated micro-extensometer with low-cost optical tracking

**DOI:** 10.1186/s12915-025-02216-9

**Published:** 2025-04-28

**Authors:** Nicola Trozzi, Wiktoria Wodniok, Robert Kelly-Bellow, Andrea Meraviglia, Aurore Chételat, Nova Adkins, Brendan Lane, Richard S. Smith, Dorota Kwiatkowska, Mateusz Majda

**Affiliations:** 1 Department of Computational and Systems Biology, John Innes Centre, Norwich, NR4 7UH UK; 2 Department of Plant Molecular Biology, University of Lausanne, Lausanne, CH-1015 Switzerland; 3 School of Biological Sciences, University of East Anglia, Norwich, NR4 7TJ UK; 4 Institute of Biology, Biotechnology and Environmental Protection, Faculty of Natural Sciences, University of Silesia, Katowice, 40-032 Poland

**Keywords:** Micro-extensometer, Plant biomechanics, Arabidopsis, Cell wall mechanics, Tissue stiffness, Optical tracking, Creep, Elasticity, Yield stress, MorphoRobotX

## Abstract

**Background:**

Plant growth and morphogenesis is a mechanical process controlled by genetic and molecular networks. Measuring mechanical properties at various scales is necessary to understand how these processes interact. However, obtaining a device to perform the measurements on plant samples of choice poses technical challenges and is often limited by high cost and availability of specialized components, the adequacy of which needs to be verified. Developing software to control and integrate the different pieces of equipment can be a complex task.

**Results:**

To overcome these challenges, we have developed a computer automated micro-extensometer combined with low-cost optical tracking (Camelot) that facilitates measurements of elasticity, creep, and yield stress. It consists of three primary components: a force sensor with a sample attachment point, an actuator with a second attachment point, and a camera. To monitor force, we use a parallel beam sensor, commonly used in digital weighing scales. To stretch the sample, we use a stepper motor with a screw mechanism moving a stage along linear rail. To monitor sample deformation, a compact digital microscope or a microscope camera is used. The system is controlled by MorphoRobotX, an integrated open-source software environment for mechanical experimentation. We first tested the basic Camelot setup, equipped with a digital microscope to track landmarks on the sample surface. We demonstrate that the system has sufficient accuracy to measure the stiffness in delicate plant samples, the etiolated hypocotyls of *Arabidopsis*, and were able to measure stiffness differences between wild type and a xyloglucan-deficient mutant. Next, we placed Camelot on an inverted microscope and used a C-mount microscope camera to track displacement of cell junctions. We stretched onion epidermal peels in longitudinal and transverse directions and obtained results similar to those previously published. Finally, we used the setup coupled with an upright confocal microscope and measured anisotropic deformation of individual epidermal cells during stretching of an *Arabidopsis* leaf.

**Conclusions:**

The portability and suitability of Camelot for high-resolution optical tracking under a microscope make it an ideal tool for researchers in resource-limited settings or those pursuing exploratory biomechanics work.

**Supplementary Information:**

The online version contains supplementary material available at 10.1186/s12915-025-02216-9.

## Background

In plants, growth and morphogenesis depend on the interaction of genetic networks, cell signaling, and mechanical forces. Turgor pressure within cells stretches the cell wall elastically, and under the influence of wall modifiers such as expansins, this tension causes the wall to expand through creep [1, 2]. Creep occurs in the presence of cell wall modifiers when tensile stress exceeds a specific yield threshold, as described in Lockhart’s growth framework [3–5]. According to this framework, growth depends on the product of extensibility factors and turgor pressure exceeding the yield point. While expansins promote creep without affecting wall elasticity [4, 6], other proteins like xyloglucan endotransglucosylase/hydrolases (XTHs) also contribute to wall extensibility [7]. On the other hand, wall stiffening can limit growth, as seen in cytokinin-induced cessation of root elongation [8]. In recent years, novel methods have been developed to measure mechanical forces in plant tissues with most observations relying on stretching tissues with an extensometer and applying localized compression to cells or specific regions using an indenter [9–11]. Extensometers quantify the stiffness of materials and how they respond to mechanical forces, allowing the evaluation of elastic and plastic behavior, stress tolerance and fracture, and structural behavior.

To measure the mechanical behavior of plant tissues, extensometers record how samples respond to applied forces. These devices quantify properties such as elasticity, creep, and yield stress of soft tissues by stretching samples while monitoring force and displacement. Strain is calculated by dividing displacement by the initial length, and stress is calculated by dividing force by the cross-sectional area, allowing a stress strain curve to be generated from the force–displacement values. The stress–strain curve normalizes force and displacement by sample dimensions, reflecting intrinsic material properties such as Young’s modulus. In contrast, the force–displacement curve represents the raw measurement data without accounting for sample geometry. The slope of the stress–strain curve over short time scales indicates the sample stiffness, which is the tissue level Young’s modulus in the direction of stretch. Creep refers to plastic deformation that occurs under constant stress over longer periods. Yield stress is defined as the minimum stress (force per unit area) required to initiate irreversible deformation. In our experiments, this is calculated by normalizing the applied force at the onset of creep by the initial cross-sectional area of the sample. Factors such as water movement in living tissues or cell wall viscoelasticity in isolated samples can affect these measurements, causing relaxation or reversible deformation. Precise control of force, displacement, and timing is essential for accurately distinguishing between plastic and viscoelastic behavior and quantifying mechanical properties.

Early extensometers often used weights to measure creep [12]. While simple and low cost, these systems lack precise control over sample deformation, making them less suitable for stiffness tests on very small samples like *Arabidopsis* hypocotyls. Modern micro-extensometers, designed for such samples, typically employ a computer-controlled actuator to displace the sample attached to a force sensor, providing precise force measurements [13–15]. While these setups are capable of accurately tracking actuator displacements, they often cannot precisely measure the sample’s deformation due to issues such as slippage at attachment points or alignment shifts as the tissue stretches. These challenges are particularly significant for smaller samples, making accurate stiffness measurements challenging without optical feedback. One solution is to mark the sample with landmarks and track its deformation using a digital camera [15, 16]. Another approach involves mounting the extensometer on a microscope with adequate resolution to use cells as deformation landmarks [17–20].

Recent extensometer setups for small biological samples often rely on specialized hardware, such as piezoelectric actuators or high-resolution linear stages, which can be prohibitively expensive for labs with limited funding or researchers exploring biomechanics on a smaller scale. For example, systems using SmarAct or Zaber actuators provide precise motion control but are typically priced beyond the reach of many academic labs. Fortunately, the decreasing costs of consumer-grade devices have made precise micro-actuators and micro-force sensors more accessible for these applications. However, software remains a significant hurdle. Many control libraries for specialized devices are proprietary and offer minimal functionality tailored to specific hardware. In addition, experimental setups frequently require separate software for each component, including the actuator, force sensor, and camera, which complicates system integration and operation.

Here, we present a Camelot system that overcomes these challenges by providing a low-cost micro-extensometer setup that is easy to assemble with minimal resources and technical expertise. The system is controlled by MorphoRobotX [15, 21–23] (www.MorphoRobotX.org), an integrated software environment for mechanical experimentation. MorphoRobotX can control a wide range of actuators, force sensors, and cameras, enabling the use of widely available components from consumer devices. An entire Camelot system, including an actuator, force sensor, control electronics, and camera achieves a resolution of approximately 5 µm. The open-source software is free to use and runs on a Linux desktop, with USB connectivity ensuring compatibility with modest hardware, including use with laptops. Its portability and suitability for high-resolution optical tracking under a microscope make Camelot an ideal tool for researchers in resource-limited settings or those pursuing exploratory biomechanics work. To put its capabilities into context, we provide Additional File 1:Table S1, which compares Camelot with other recent micro-extensometer systems used in plant biomechanics, showing differences in actuation, force sensing, deformation measurement, software integration, and cost.

## Results

### System overview

**Fig. 1:**
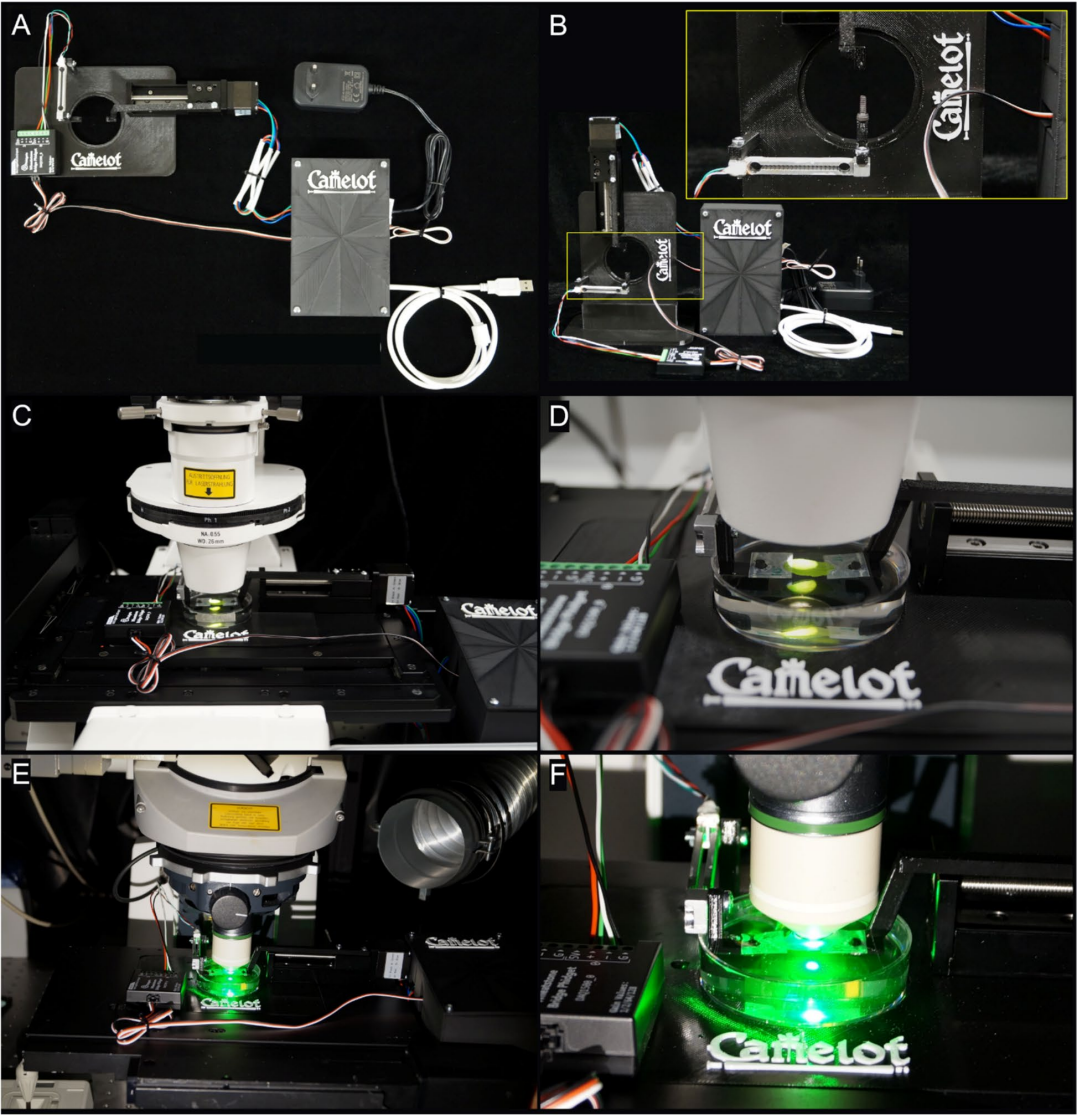
Fully integrated experimental setup for simultaneous mechanical measurement and imaging under a confocal microscope.** A** Overview of the assembled system, including the linear motion stage, the force sensor connected to the Wheatstone Bridge Phidget, with both connected to the VINT Hub Phidget to convert to USB. Electronic components are enclosed in a custom 3D-printed housing for protection and organization. The system interfaces with a computer via USB for real-time data acquisition and control. **B** Vertical configuration of the force sensor for calibration. The inset highlights the force sensor mounted in a custom 3D-printed holder with precise alignment for mechanical measurements. This configuration allows accurate force calibration while maintaining stability during operation. **C** Integration with a confocal microscope, with the baseplate securely mounted on the microscope stage to align the force sensor and sample with the optical path, enabling simultaneous imaging and mechanical testing. **D** Close-up of the sample mounted on the force sensor under the confocal microscope objective, positioned in a Petri dish for hydration and optical compatibility. **E** Confocal imaging of the sample under green fluorescence, showing alignment within the optical path. **F** Magnified view of the same sample under green fluorescence, illustrating finer details and secure alignment between the force sensor and the Wheatstone Bridge Phidget

The Camelot micro-extensometer system consists of three primary components: a force sensor with a sample attachment point, an actuator with a second attachment point to stretch the sample, and a camera to monitor deformation (Fig. 1A;Additional File 2: Fig. S1). The force sensor, or load cell (Additional File 2: Fig. S1 C-D), positioned on the linear motion stage (Fig. 1A;Additional File 2: Fig. S1 K), is a parallel beam sensor commonly used in digital weighing scales, optimized for single-axis force measurement with minimal sensitivity to off-axis forces. Similar sensors were utilized by Bidhendi et al. [14] and are available in various ranges, with a maximum force of several kilograms down to 10 g. The 10 g and 100 g models in our system cost approximately 10 GBP each. The 10 g sensor offers a resolution of approximately 10 μN, making it highly suitable for precise biomechanical measurements on small samples (Additional File 2: Fig. S1 C-D). For calibration, the force sensor is configured vertically using a custom 3D-printed holder for precise alignment and stability during force calibration (Fig. 1B,Additional File 2: Fig. S1 N, Q). Phidgets or “Physical Widgets” provide an accessible interface for hardware control, analogous to software widgets in a graphical user interface [24]. Multiple Phidgets, including those for force sensor and actuator control, can be connected to a single Phidget Hub via USB (Additional File 2: Fig. S1I), facilitating computer integration. The sensors use a Phidgets Wheatstone Bridge to measure small resistance changes caused by deflection. With a DC input of 5–10 V, they generate an output voltage of 2–10 mV per input volt, directly proportional to the applied load. This low output voltage is subsequently amplified to a range suitable for an analog-to-digital converter (typically 0 to 5- or 10-V DC). Consistent with Bidhendi et al. [14], we use a Phidgets bridge amplifier to power the sensor and amplify the signal (Additional File 2: Fig. S1H). The system's modular design, featuring a Wheatstone Bridge and compatibility with various load cells, including S-beam sensors from Futek (www.futek.com) [13, 19], allows seamless integration with diverse optical components, such as C-mount microscope cameras used in confocal setups. For these setups, the baseplate is securely mounted on the microscope stage, aligning the force sensor and sample with the optical path for simultaneous imaging and mechanical testing (Fig. 1C). The baseplate accommodates a Petri dish for sample hydration (Fig. 1D) and the setup enables confocal imaging to observe deformation at cellular resolution during mechanical testing (Fig. 1E–F).

The linear actuator in our system uses a stepper motor that drives a screw mechanism to move a stage along linear rail (Additional File 2: Fig. S1 A, B). Commonly used for precise movements in applications like CNC (Computer Numerical Control) machine control, these actuators are readily available in various sizes. Our setup features a compact actuator with a 1-mm pitch screw, enabling 1 mm of movement per full motor revolution. The stepper motor rotates in 1.8° increments, providing a movement resolution of 5 μm per step, which meets the requirements of most experimental applications. For even finer control, the Phidgets stepper motor controller can subdivide these steps as needed (Additional File 2: Fig. S1 J). The stepper motor follows the NEMA 11 standard, which specifies a 28-mm square faceplate, making it a compact and widely compatible choice. This standardization ensures easy assembly and interchangeability with components from different manufacturers. The uniform color coding of control wires further facilitates integration. The linear actuator selected for our setup has good build quality at a cost of approximately 50 GBP. It is durable, with no noticeable looseness in its mechanism, and has proven to be accurate and dependable for our experimental applications.

**Fig. 2:**
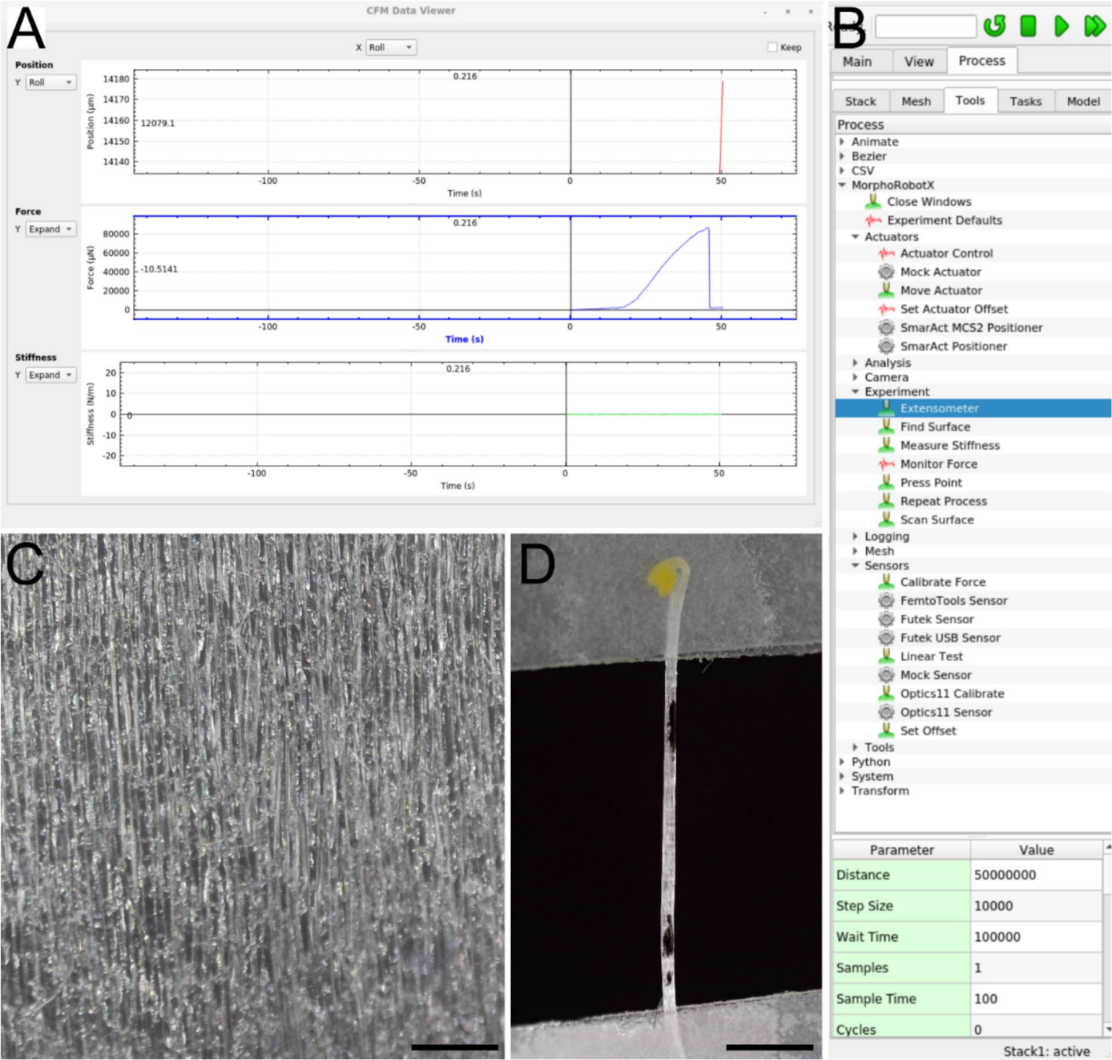
Overview of the MorphoRobotX interface and samples under tension. **A** The pop-up window (CFM Data Viewer) relative to the Extensometer process, showing the force curve generated during a stretching experiment, illustrating the force applied to the sample until rupture. **B** The MorphoRobotX interface menu displays all the necessary setup, calibration, and experiment execution processes. **C** Onion epidermal layer cells captured through the setup camera, showing the sample under tension without visible deformation. **D** Etiolated 3-day-old Col-0 hypocotyl, attached to Tough-Tags with visible landmarks marked on the sample. The sample is shown at its ultimate stress point just before rupture, illustrating deformation under tension. Scale bars: 1 mm (**C**); 2 mm (**D**)

The third component of the system is the camera, which integrates with the MorphoRobotX interface to capture deformation data. The software interface displays the menu for setup, calibration, and experiment execution, while a pop-up window shows force curves generated during stretching experiments, illustrating the force applied to the sample until rupture (Fig. 2A, B). We use a compact USB digital microscope, with models providing a resolution of 2592 × 1944 pixels available for approximately 100 GBP. At maximum magnification, the camera can visualize individual plant cells, but lower magnification is typically used to capture the full tissue section along with landmark dots, as demonstrated for onion epidermal cells and hypocotyl samples under tension (Fig. 2C, D). The camera operates independently of the micro-extensometer setup, allowing the system to be conveniently used under a conventional microscope when required. Our software interfaces with the digital microscope using the standard Linux webcam driver and is compatible with a range of smaller digital microscope cameras. Through Linux libraries such as OpenCV (opencv.org) and Micro-Manager (micro-manager.org), support is provided for various microscope cameras (typically C-mount), which are used in experiments requiring higher resolution or specialized imaging. Additional software libraries provided by camera manufacturers, such as the IDS-Peak library, are installed for some experiments to enable compatibility with specific cameras that are not accessible through OpenCV or Micro-Manager. Since MorphoRobotX uses a plugin-based system for camera drivers (processes), it is possible to integrate any camera that can be accessed through Linux libraries. We provide Debian packages for Ubuntu 20 and 22, which have also been verified to work on Windows using Windows Subsystem for Linux (Additional File 3:Dataset).

### Basic setup with digital microscope

**Fig. 3:**
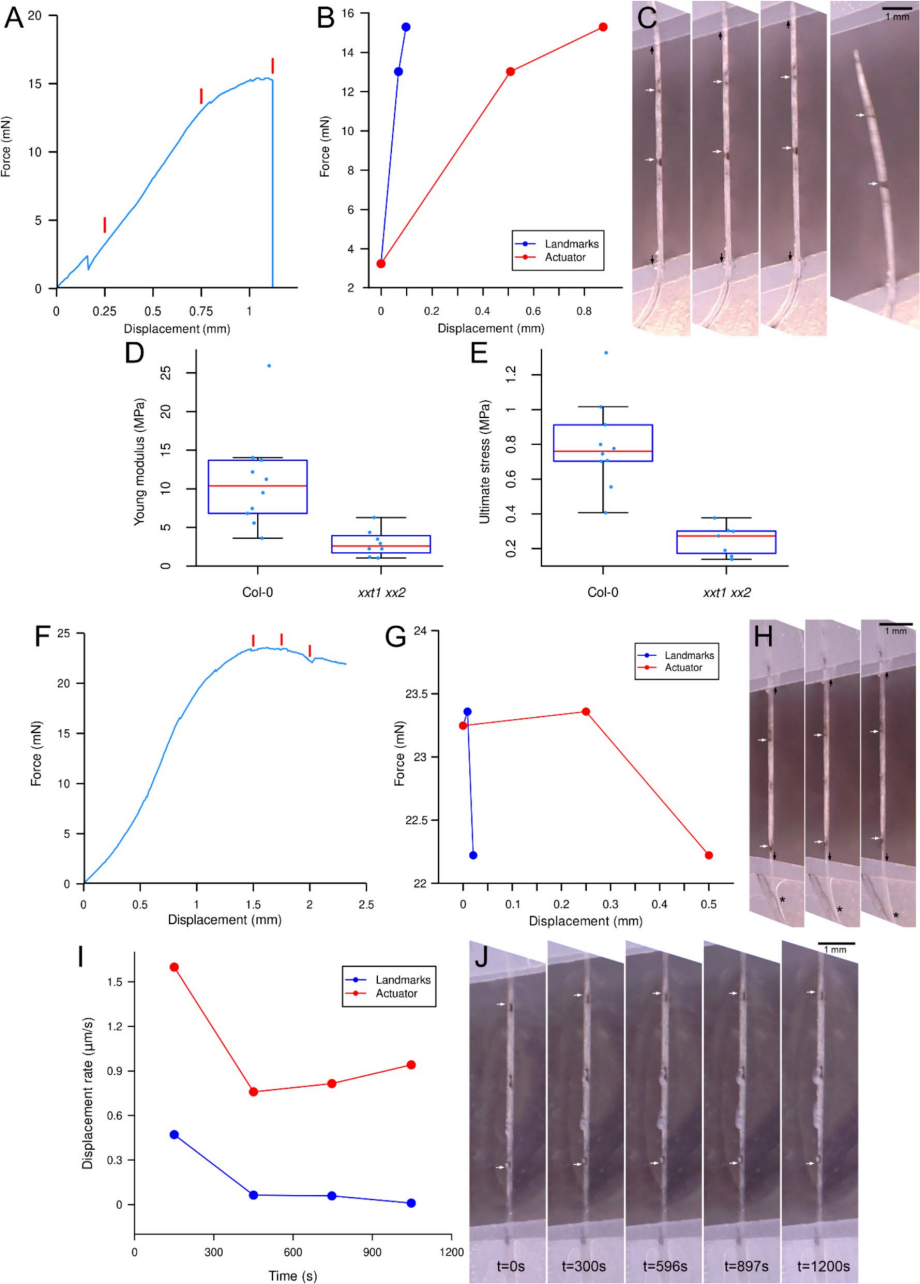
Mechanical properties and deformation analysis of etiolated Arabidopsis hypocotyls under tension and creep experiments.** A–C** Exemplary force–displacement curve (**A**), force plotted versus actuator or landmark-assessed displacement (**B**), and corresponding hypocotyl images (**C**) from an experiment ending in breakage. Three red segments in **A** mark the points corresponding to measurements in **B** and the first three images in **C**; the final image is captured immediately after breakage. The force increase between the first two segments and the strain from the first two images were used to compute Young’s modulus. Landmark-based strain (white arrows) was 2.09% for the first image pair and 0.88% for the second, whereas sticker-based strain (black arrows) was higher (5.90% and 4.57%) due to slippage. **D,E** Young’s modulus (**D**) and ultimate stress (**E** for Col-0 and *xxt1 xxt2* hypocotyls are shown; in the boxplots red lines denote the median, boxes span the first and third quartiles, whiskers extend to values within 1.5 × IQR, and dots represent individual measurements. Col-0 (*n* = 10) differs significantly from *xxt1 xxt2* (*n* = 8 for modulus; *n* = 7 for stress) (*t*-test,* p* = 0.0031 and *p* = 0.00007). **F–H** Exemplary force–displacement curve (**F**) with force plotted versus actuator or landmark-assessed displacement (**G**) and corresponding hypocotyl images (**H**). In the final stage, indicated by three red segments in **F** corresponding to the measurements in **G** and images in **H**, grip displacement mainly caused sample slippage with negligible hypocotyl strain. Landmark-based strain (white arrows) is 0.28% for the first image pair and 0.36% for the second, whereas sticker-based strain (black arrows, region marked by black asterisks) is 5.19 and 4.17%, respectively. **I,J** Exemplary creep experiment results from the basic setup. **I** Displacement rates for landmarks (blue) and actuator grips (red) plotted over time, computed over 300-s intervals from landmark positions (white arrows) or actuator positions recorded in a.csv file. **J** Corresponding hypocotyl images

The basic Camelot setup was equipped with a compact digital microscope to track landmarks on the sample and Young’s modulus, and breaking stress of etiolated *Arabidopsis* hypocotyls was measured. The experiments were conducted on wild-type (Col-0) and *xylosyltransferase1 xylosyltransferase2* (*xxt1 xxt2*) double mutant, which lacks xyloglucan in the cell walls [25]. This allowed us to compare results obtained with Camelot to previously published data using alternative setups [25, 26]. Prior to measurements, landmarks were drawn on the surface of the hypocotyls using an India ink marker (Faber-Castell Pitt Artist Pen Brush, Black 199***). These landmarks were positioned a small distance from the fixing tags as far apart as possible to reduce measurement noise from the images (larger distances are easier to quantify). Where gradients of stiffness are to be interrogated, one could place a series of dots in-between. Before the experiment, three hypocotyls from each batch were imaged using a stereomicroscope (Leica M60) equipped with a digital camera (IC80 HD). These images were analyzed in ImageJ to measure the hypocotyl diameter, which was then used to calculate the cross-sectional surface area assuming a cylindrical shape for the hypocotyl. Next, each hypocotyl was mounted between two transparent stickers (NIIMBOT Thermal Labels, Transparent Stickers, 14 × 30 mm). One sticker secured the apical portion of the hypocotyl, including the cotyledons, while the other held the root and basal portion of the hypocotyl. After positioning either the cotyledon or root pole of the hypocotyl between the two halves of a folded sticker, each sticker was punched using an office puncher and mounted onto the pins connected to the load cell or actuator. Hypocotyl images captured after each stretching step (Additional File4:Movie 1), along with corresponding force readings, were used to calculate Young’s modulus and ultimate stress for each sample (Fig. 3A–C). For each hypocotyl, a nearly linear section of the force–displacement curve was identified (see the curve fragment between the first and the second red line segments in Fig. 3A). Kinks observed at the start of stretching are due to initial sample movement, and although our analysis considers only the curve segments recorded after these initial movements have stabilized, optical tracking eliminates this problem. It is also important that the section chosen for analysis is consistent between samples and is before the sample softens as it approaches failure. On the other hand, the larger the section, the easier it is to determine the deformation from the optical landmarks. The chosen section spanned 50 steps, corresponding to a displacement of at least 0.25 mm, to minimize errors in strain assessment and allow for reliable calculations of material properties. Using ImageJ, we measured the distance between landmarks in images corresponding to the beginning and end of the selected portion of the force–displacement curve (Fig. 3B,C). The relative distance increment between landmarks was used to calculate the sample strain. The corresponding increase in stress was calculated as the applied force divided by the cross-sectional area of the hypocotyl, which was measured at the beginning of the experiment. While the effective cross-sectional area may decrease slightly during stretching due to the Poisson effect, assuming a constant area is a standard approach in stress–strain analysis. These reductions are typically small and do not significantly impact stress calculations. The changes in stress and strain between extensometer steps in the region of the curve were used to compute Young’s modulus, which approximates this section as linear. For the same samples, the ultimate stress at the point of sample rupture was determined from the location of a sudden drop in force on the force–displacement curves (Fig. 3E). The results of these analyses, performed with the basic Camelot setup, show consistent and statistically significant differences in Young’s modulus and ultimate stress between the etiolated hypocotyls of *Arabidopsis* Col-0 and the *xxt1 xxt2* mutant (Fig. 3D) and are consistent with previous studies [25] where it was reported that the *xxt1 xxt2* mutant exhibits altered mechanical properties in its cell walls, including reduced tensile strength and stiffness. These results demonstrate that basic Camelot set up is sufficient to produce data showing mechanical differences between two genotypes using simple pen dots as landmarks to measure displacement.

Close examination of hypocotyl images captured during the measurements enabled us to distinguish between actual sample stretch and sample slippage from the grips (stickers), allowing for an accurate assessment of sample strain. This detailed imaging also facilitated a critical interpretation of the force–displacement curves. For instance, the curve shown in Fig. 3F suggests sample relaxation toward the end of the experiment. However, inspection of the images reveals that the observed decrease in force was primarily due to significant slippage of the hypocotyl from the stickers (Fig. 3F–H).

Using etiolated hypocotyls of wild-type *Arabidopsis*, we also conducted a creep experiment to evaluate the time-dependent deformation of the samples (Fig. 3I,J). As anticipated, the rate of sample creep, assessed based on the positions of landmarks, decreased over time (blue curve in Fig. 3I). However, it is important to note that the rate of grip displacement during the experiment was higher and increased rather than decreased in the later stages. This behavior was attributed to significant sample slippage from the grips (red curve in Fig. 3I).

### Setup using C-mount microscope camera

**Fig. 4:**
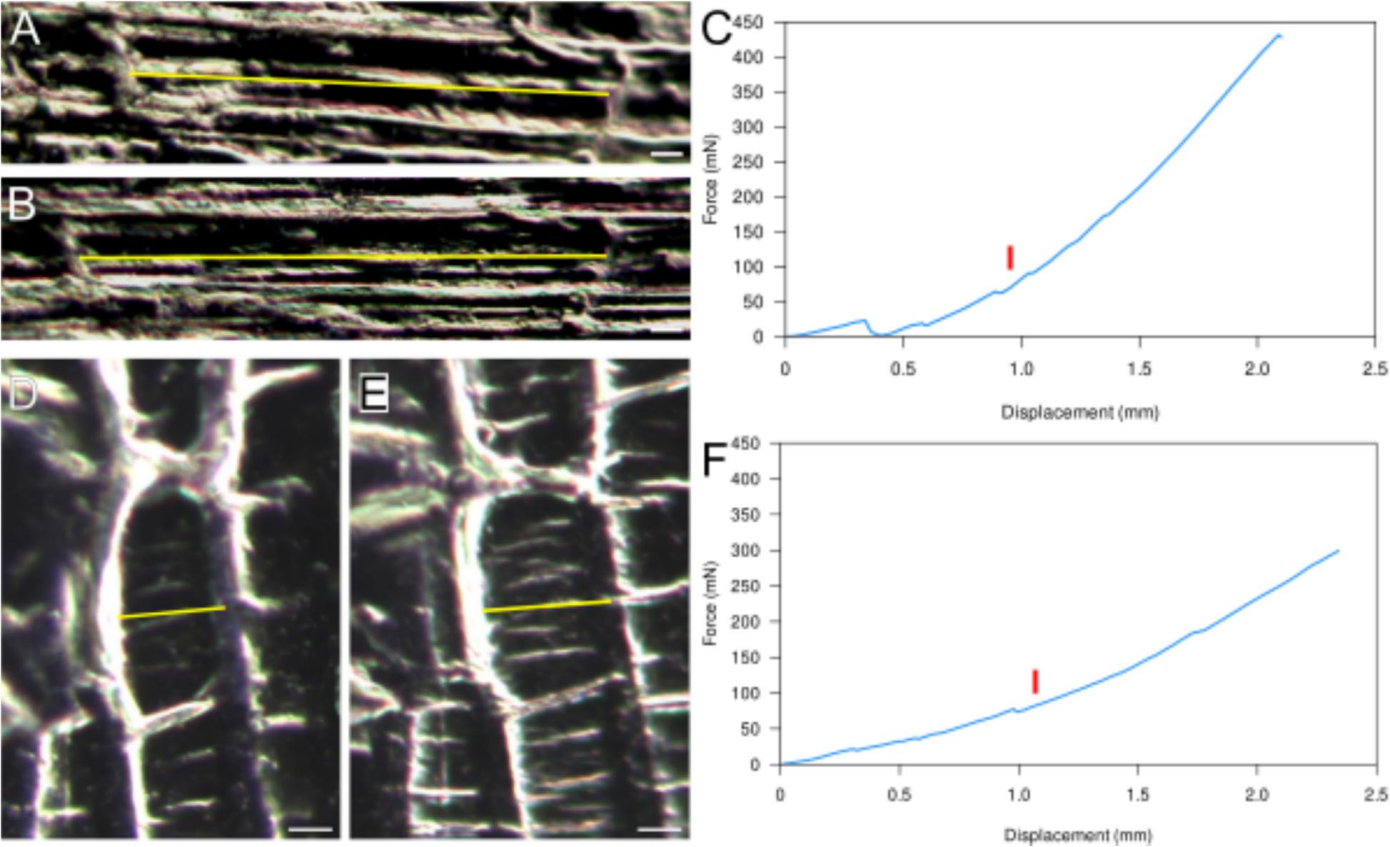
Onion epidermal peel deformation in extensometer experiments can be accurately measured using cell junctions. **A,B** Longitudinally stretched cell in a relaxed state (**A**) and under 10% strain (**B**). Yellow line highlights cell junctions where distances were measured from. **C** Corresponding force–displacement curve for longitudinally stretched sample. **D,E** Transversely stretched cell in a relaxed state (**D**) and under 10% strain (**E**). Yellow line highlights cell junctions where distances were measured from. **F** Corresponding force–displacement curve for transversely stretched sample. The red lines in **C** and **F** mark the onset of plastic deformation, as determined based on cell junction displacements. Scale bars: 20 µm

A more accurate measure of deformation can be obtained by analyzing the cells or cell junctions as landmarks, using an inverted light or fluorescence microscope with a C-mount camera. For this setup, we placed Camelot on an inverted microscope (Axiovert 35 M, Zeiss, Germany) with a C-mount camera (U3 - 3280SE, IDS, UK) to provide optical tracking. The IDS camera is just one example of a CCD camera that can be controlled by MorphoRobotX, synchronizing image capture with each step in the stepper motor. We stretched onion epidermal peels in both longitudinal and transverse directions (Fig. 4) to test if similar results could be obtained to previously published data [15]. Onion epidermal peels 4 mm wide were prepared as described in Majda et al. (2022). Longitudinally stretched samples were mounted so that the direction of stretch was axial along the tissue and transversely stretched samples were mounted so that the direction of stretch was circumferential. Distance between cell junctions was measured in ImageJ to accurately determine strain.

Longitudinally stretched tissues reached a stress of 1.19 MPa at 10.6% strain and withstood a maximum force of around 430 mN before breakage. Transversely stretched tissues reached a stress of 2.08 MPa at 19.0% strain and withstood a maximum force of around 300 mN before breakage. At 10% strain, longitudinally and transversely stretched samples had Young’s moduli of 11.23 and 6.20 MPa, suggesting that the tissue is 1.94 times stiffer longitudinally than transversely, which is comparable with previous results [15]. The red lines in Fig. 4C and F mark the onset of plastic deformation, which was identified based on irreversible displacements of cell junctions. We defined the elastic region as the portion of the force–displacement curve beyond initial artifacts as the sample transitions from a non-tensioned to a tensioned state, but before irreversible deformation. For the longitudinally stretched tissue, the distance in cell junctions increased from 147 to 175 µm, giving a strain of 19.1%, whereas the actuator had moved from 730 to 1920 µm, overestimating strain at 163%. For the transversely stretched tissue, the distance between cell junctions increased from 318.9 to 352.8 µm, giving a strain of 10.6%, whereas the actuator had moved from 530 to 1110 µm, giving a strain of 109.4%, more than an order of magnitude higher than the actual tissue deformation. This confirms that displacement-based strain measurements without optical tracking can be highly inaccurate. The discrepancy between actuator-based and optical strain measurements arises from a combination of sample slippage and fixation flexibility. In our experiments, actuator displacement consistently overestimated strain relative to cell junction tracking. While some variation is expected due to differences in measurement resolution and local strain distribution, optical tracking provides a more reliable measure of tissue deformation.

We also found that the resolution and magnification are sufficient to capture mechanical failure at the cellular resolution. We could see that when a sample fails, the tissue separation occurred within a cell and propagated across the tissue. Thus, Camelot coupled with a top-mounted CCD can capture extensometer experiments with cellular resolution for accurate optical tracking.

### Confocal extensometer

We used the confocal extensometer to analyze the deformation of epidermal cells from *Arabidopsis* leaves during stretching experiments. To facilitate tracking of the cell outlines, the plasma membrane marker line (*pUBQ10::acyl-YFP*) [27] was used. The samples were mounted onto Camelot’s extensometer arms using Tough-Tags and submerged in water within a small Petri dish to prevent desiccation.

**Fig. 5:**
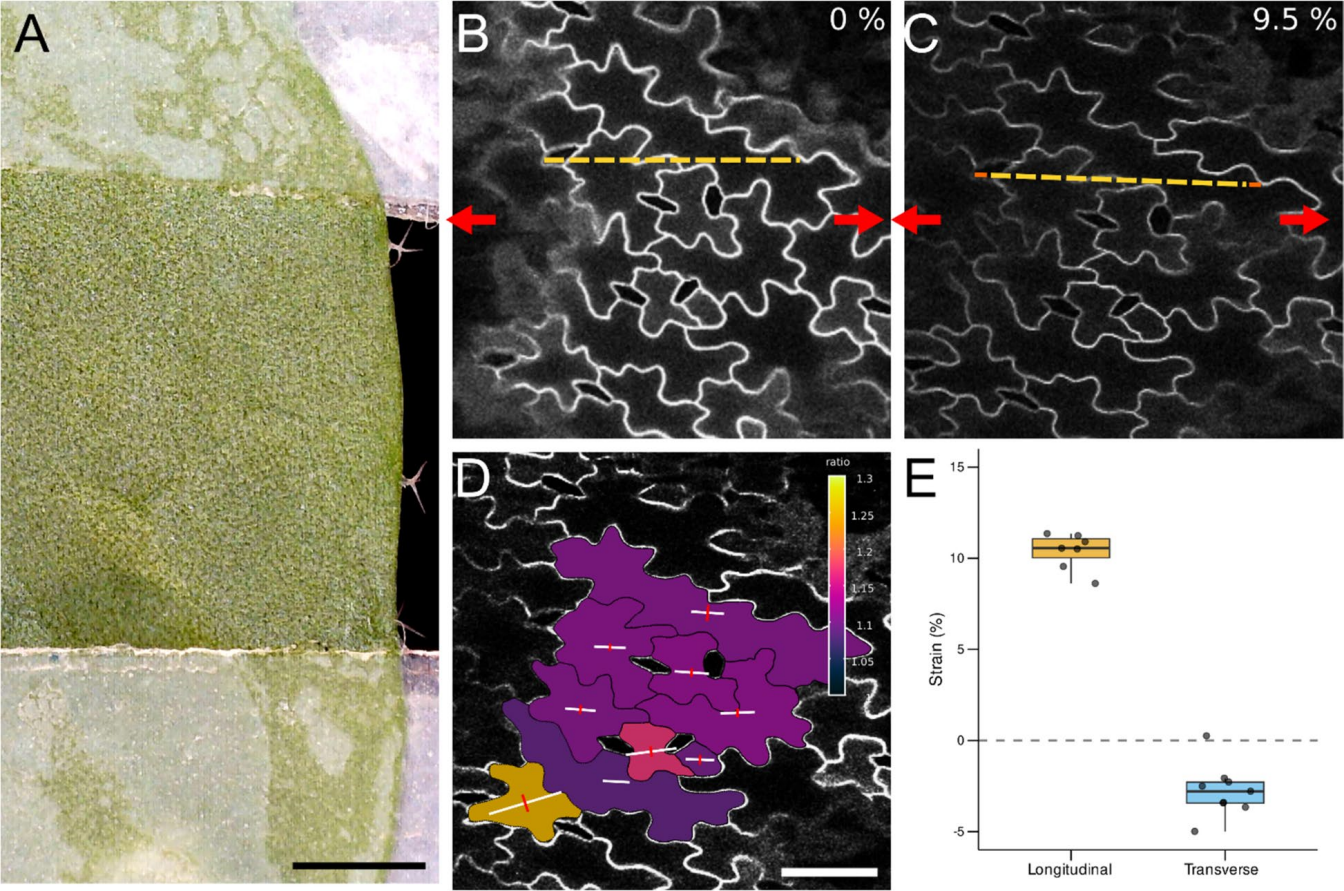
Cellular deformation analysis on Arabidopsis leaf. **A** Arabidopsis leaf sample attached to tags prior to stretching. Scale bar: 1 mm. **B,C** Confocal z-stack images of abaxial leaf cells before stretching (**B**) and at 9.5% strain or maximum deformation before rupture (**C**). Arrows indicate the stretching direction. **D** Heatmap of cell deformation of selected cells. Deformation crosses are calculated using MorphoGraphX, with white arms indicating extension and red arms indicating contraction, visualized at the cell centroids. Arrows indicate the stretching direction. **B**, **C**, and **D** share the same scale bar of 50 µm. **E** Boxplot showing longitudinal and transverse strain (%) of the selected cells in **D**. Longitudinal strain corresponds to the stretching direction, while transverse strain is perpendicular to it. The grey dashed line at 0% indicates no change in cell size

The setup was coupled with an upright Zeiss LSM 710 NLO confocal microscope, operated in single-photon mode. Confocal z-stack images were acquired at three stages: before stretching, during incremental deformation, and immediately prior to rupture (Fig. 5, Additional File 2: Fig. S2, Additional File 5: Movie 2). These images were processed in MorphoGraphX [28] to compute the principal directions of deformation. Deformation was visualized at the cell centroids, with white lines indicating extension and red lines indicating contraction, with the lengths proportional to the amount.

During the stretching experiments, cells exhibited anisotropic deformation, characterized by elongation along the axis of applied force and contraction perpendicular to it. This behavior is consistent with the concept of Poisson’s ratio, which describes the ratio of transverse strain to axial strain in materials under stress. In plant cells, Poisson’s ratio typically ranges from 0.18 to 0.30 [29], reflecting the lateral contraction that accompanies axial stretching, although that is for the cell wall itself, and here a cellular tissue is being stretched.

## Discussion

The Camelot system offers a simple and cost-effective solution for mechanical testing of small to medium-sized biological samples. It operates entirely on open-source software and is assembled from readily available consumer components. The cost and complexity of extensometer systems are largely influenced by the actuator choice. For instance, systems described by Schluck et al. [18], Hofhuis et al. [13], and Robinson et al. (2017) use SmarAct (www.smaract.com) piezo-based stick–slip positioners, which deliver nanometer resolution but have significant drawbacks. These high-quality positioners are costly, priced between 6000 and 9000 GBP depending on configuration, with long lead times and high sensitivity to dirt and shocks, making them complex to program and handle. A more affordable alternative involves high-precision screw-type linear stages from Thorlabs [14] or Zaber [30], costing around 1350–2000 GBP. While these actuators provide micrometer resolution, they remain expensive and may lack Linux driver support, as in the case of Thorlabs. Camelot uses a low-cost (50 GBP) screw drive actuator with an estimated 5 µm resolution sufficient for most sample testing. For example, achieving 5% deformation on a 5-mm sample would result in 50 steps. Although less precise than higher-end actuators, this resolution generally meets most experimental needs. Relying solely on actuator displacement for tissue deformation measurement is impractical because it includes errors from sample slippage, rotation, and mounting tag flexibility, especially in smaller samples. A significant consequence of relying on the actuator position is overestimation of the strain, which leads to underestimation of Young’s modulus. Therefore, we measure strain using synchronized images to track sample landmarks, which provides a direct and accurate assessment of tissue deformation. This approach, implemented in Camelot, improves the reliability of biomechanical measurements. Thus, effective resolution depends on the accuracy of image-based landmark measurements rather than actuator resolution. To further minimize slippage, adhesives such as cyanoacrylate-based glues [19] can be employed. For sturdier tissues, mechanical clamps may be more appropriate. However, in biological samples, it is unlikely that slippage can be entirely eliminated with improved fixation.

To demonstrate the utility of the device, we performed experiments on living plant tissue in several configurations. In our most basic Camelot set up with a digital microscope, we were able to determine the tissue Young’s modulus and breaking stress of wild-type and *xxt1 xxt2* double mutant etiolated *Arabidopsis* hypocotyls. We tracked deformation using landmark points marked on the hypocotyl using a permanent marker from synchronized images recorded by the software. We found that Young’s modulus for wild-type was around five times higher than *xxt1 xxt2*, and that ultimate stress at breaking for wild-type was more than double that of *xxt1 xxt2*. This demonstrates that a complete Camelot system costing under 500 GBP can measure similar biomechanical differences to those previously reported [25] in small and delicate plant samples. We also used the system to perform creep experiments that showed significant creep in the first 5 min that tapered off over time. This demonstrates that a budget Camelot system can be used to perform a range of the most common extensometer experiments.

For labs that have access to microscopes with CCD cameras, we show that cellular-level deformations can be tracked using mostly the same Camelot hardware. We used an IDS camera mounted to an inverted light microscope, but other cameras, microscope and fluorescence combinations can be also accommodated. Our data showed that onion epidermal peels are stiffer longitudinally than transversely, in line with previously published results [15].

Camelot can also be combined with confocal microscopy [18–20] by adapting the mounting board. This can be used to measure cell deformation and response under stress. An *Arabidopsis* leaf was placed under a measured strain and the deformation of individual cells in a tissue was determined using MorphoGraphX to analyze their shape change. A combination of Camelot with confocal microscopy allows for precise segmentation and tracking of deformation at the individual cell level by using cell boundaries. However, the slower imaging speed of confocal microscopy can lead to partial sample relaxation between steps. Additionally, images must be synchronized manually with the Camelot system, as most commercial confocal setups currently lack open software integration to directly trigger image acquisition.

The Camelot system provides a low-cost, accessible, open-source solution that can be built from widely available consumer components, adaptable for various experimental needs. Several potential improvements could enhance the system’s capabilities. While the low-cost actuator’s resolution and precision is sufficient for many biomechanical measurements, a higher-end device might be beneficial in specific scenarios, such as oscillatory loading/unloading [31], or precise force applications where actuator backlash could interfere. High-quality actuators, such as SmarAct models, produce less vibration, which could be an issue for confocal applications. The MorphoRobotX software already supports SmarAct and Zaber stages, with options to integrate other types of actuators that have Linux drivers.

Given the importance of accurate deformation tracking, another key improvement could involve automated landmark recognition, potentially through AI-based methods. This feature would allow users to select landmarks at the start of the experiment, with the software tracking the landmarks as the sample stretches, enabling automatic calculation of deformation for each step. Additionally, a movable camera stage could keep landmarks within the field of view, facilitating greater zoom and higher resolution. However, implementing these enhancements would add considerable complexity and cost to the system.

## Conclusions

This work demonstrates how an affordable and adaptable extensometer like Camelot opens new possibilities for biomechanical research. By combining consumer hardware with open-source control software, it lowers the barrier to precise mechanical measurements. Its compatibility with a range of imaging platforms allows accurate quantification of tissue deformation at multiple scales. Camelot offers a practical path for deeper exploration of the mechanical forces driving biological growth and development, even in resource-limited settings.

## Methods

**Fig. 6:**
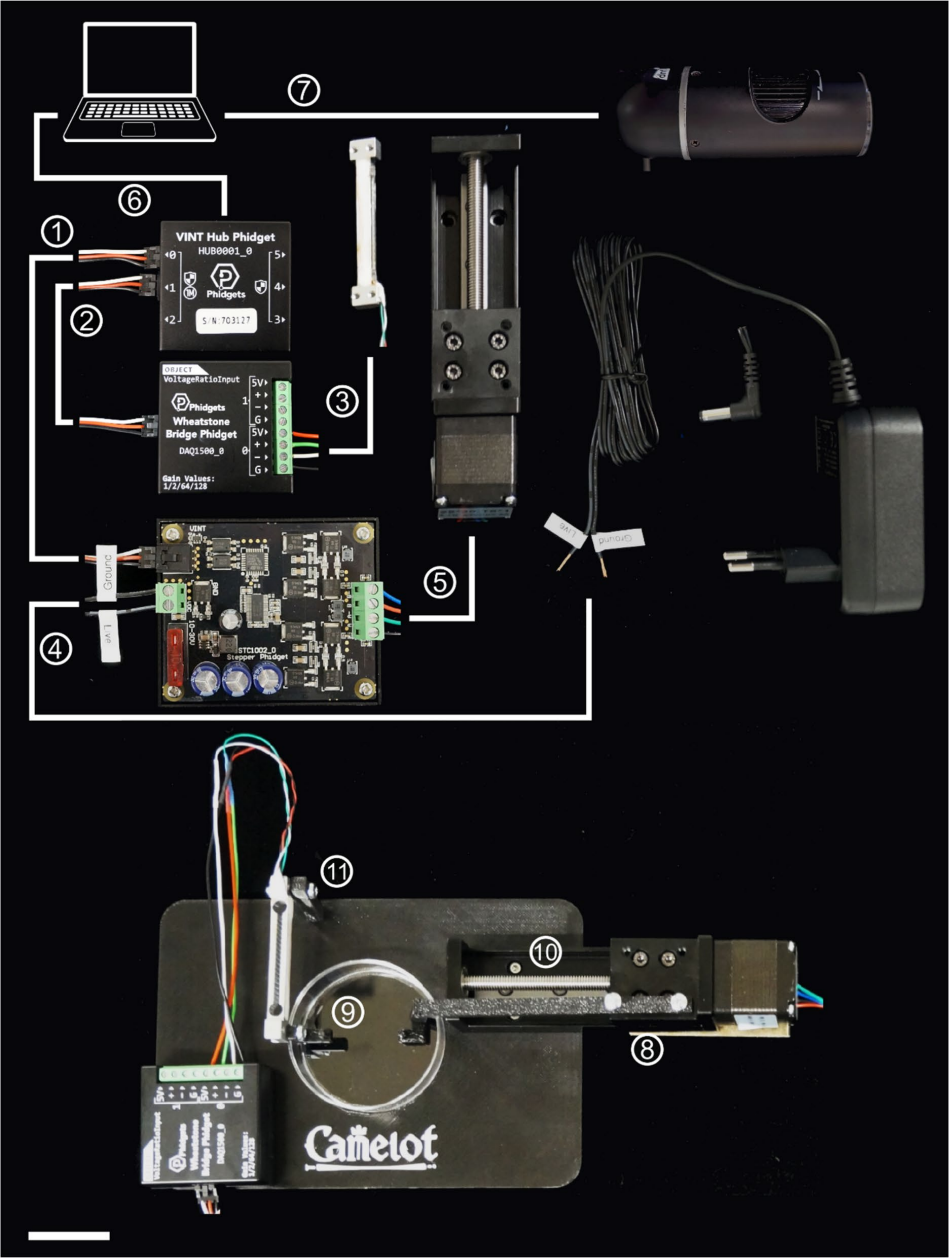
Assembly of the Camelot micro-extensometer system. (*1*) Connect the VINT Hub Phidget (HUB0001_0) to the Stepper Motor Phidget (STC1002_0) with a Phidget cable. (*2*) Connect the VINT Hub Phidget to the Wheatstone Bridge Phidget (DAQ1500_0) with a Phidget cable. (*3*) Attach the force sensor (Phidgets 3133_0 Micro Load Cell) to the Wheatstone Bridge via a wiring harness, match wiring colors, red for power, black for ground, and green and white for signal at the screw terminals. (*4*) Connect the Stepper Motor Phidget to the power supply (Mean Well GST25 A05-P1 J, 12 VDC, 2 A) with the correct polarity. (*5*) Wire the actuator (Befenybay 50 mm NEMA11 T6 × 1) to the Stepper Motor Phidget following the coil configuration (A +, A −, B +, B −). (*6*) Connect the VINT Hub Phidget to the computer using a Micro-USB-to-USB cable for data acquisition and control. (*7*) Attach the camera to the computer via USB for image-based strain tracking. (*8*) Secure the actuator stretching arm to the actuator. (*9*) Mount the stretching pin for mounting samples to the force sensor. (*10*) Fasten the actuator to the Camelot stage with screws. (*11*) Fix the force sensor to the Camelot base, routing the wiring to avoid sharp bends. This setup synchronizes actuator movement, force measurement, and optical tracking within the MorphoRobotX software. Scale bar: 30 mm

The Camelot system is composed of modular 3D-printed components designed for mechanical testing of biological samples (Additional File 2: Fig. S1 K-Q, Additional File 2: Fig. S3). These components include the linear motion stage with a central hole for illumination, Petri plate positioning, and actuator mounting (Additional File 2: Fig. S3 A, B,Additional File 2: Fig. S4B), as well as a calibration stage with a central gap to securely hold the setup during calibration procedures (Additional File 2: Fig. S3 C,Additional File 2: Fig. S4E-F). The arms of the micro-extensometer, equipped with pins for mounting samples using adhesive tape, ensure stable attachment to both the actuator and the sensor (Additional File 2: Fig. S3D, E). Additional structural components, such as the electronics box, protect the stepper controller and hub while providing sufficient ventilation and cable routing space (Additional File 2: Fig. S3 F-H, Additional File 2: Fig. S4 C-D). Collectively, these parts can be seamlessly assembled into a fully functional setup for calibration and micro-extensometer experiments (Fig. 1), as shown in the detailed assembly steps in Fig. 6, with the relative information for each component in Table 1. The Camelot setup can be configured for different experimental needs. For example, the basic configuration with a digital microscope enables straightforward mechanical measurements with high-resolution imaging (Additional File 2: Fig. S5 A, B). Alternatively, the system can be mounted on an inverted microscope for cellular resolution (Additional File 2: Fig. S5 C-E). Each setup is optimized for its specific purpose, whether focusing on external deformation measurements or high-resolution observations of tissue and cellular behavior. Assembly of the system begins with connecting the Wheatstone Bridge, sensor, and actuator to the Phidgets Hub and ensuring proper data flow to the computer (Fig. 6). The modular design allows mounting onto either a 3D-printed Camelot baseplate or a DIY plastic base created by drilling a plastic sheet to accommodate the setup (Additional File 2: Fig. S5). Additionally, detailed 3D-printing parameters are provided in Table 2, with.stl files available for download in Additional File 3: Dataset, making the system accessible and reproducible. These files can be easily and inexpensively uploaded to online manufacturing services, allowing users to have the components professionally fabricated with minimal effort.

**Table 1:** Componentsand costs for the Camelot setup. A detailed breakdown of the parts required for the Camelot setup, excluding 3D-printed components. Costs are provided in GBP (£)

**Part**	**Make**	**Model**	**Cost (GBP)**
Linear actuator with motor	Befenybay	50 mm NEMA11 T6 × 1	47
Camera (2592 × 1944)	Celestron	44308	97
10 g load cell	Dongguan Science & Tech	10 g	8 + 16^a^
100 g load cell	Phidgets	139_0	8
Stepper motor controller (8 A)	Phidgets	STC1002_0	82
Wheatstone Bridge	Phidgets	DAQ1500_0	30
VINT Hub	Phidgets	HUB0001	30
Power supply (12 Vdc, 2 A)	Phidgets	3025_0	12
Mini-USB Cable (180 cm)	Phidgets	3018_0	2
10 cm cable (× 2)	Phidgets	3003_0	3
2-axis manual stage^b^	Hyuduo	40 × 40	90
3D-printed components^c^	Generic	-	9
Nuts, bolts, small hardware	Generic	-	20
**Total**			**438**

^a^Ordered from Alibaba, shipping (16 GBP) costs more than a sensor (10 GBP), best to order multiples

^b^This stage is optional but helps to position the sample precisely. Any suitable stage can be used, for example one recycled from an old light microscope

^c^A home-made plastic base can also be used as an alternative to 3D-printing

**Table 2:** 3D-printed components and costs for the Camelot setup. A detailed breakdown of the 3D-printed components required for the Camelot setup (Additional File 3: Dataset). Costs are provided in GBP

Component	Material	Filament	Infill (%)	Supports	Rotation ($^\circ$)	Filament use (g)	Printing time (h)	Cost (GBP)
Linear motor stage	PETG	Matte black, matte white	20	Build plate	-	88.3	3.2	2.36
Calibration stand	PETG	Matte black	20	Build plate	-	86.5	3	2.30
Electronics box	PLA	Matte black	20	Everywhere, tree	-	102	3.6	2.04
Box lid	PLA	Matte black	20	Build plate	Y: 180	121.2	3.7	2.15
Actuator arm	PETG	Matte black	40	Everywhere, tree	Y: 180	3.1	0.5	0.09
Sensor arm	PETG	Matte black	80	Everywhere, normal	-	0.7	0.3	0.02
Stage holder	PETG	Matte black	20	-	-	202.8	6.5	5.35
**Total**						**604.5**	**21**	**14.31**

The linear motor stage of the Camelot system integrates with advanced imaging platforms, including confocal, two-photon, and epifluorescence microscopes, to enable simultaneous mechanical measurements and high-resolution imaging. As shown in Additional File 2: Fig. S6, the force sensor and sample holder align within the optical path, ensuring positioning under the confocal objective lens (Additional File 2: Fig. S6 A-B). The stability provided by the “Camelot” baseplate supports reliable imaging and mechanical testing without interference from sensor wiring (Additional File 2: Fig. S6 C-D). The system demonstrates versatility in capturing fluorescence signals at different wavelengths, using green and blue laser illumination to produce clear, interference-free images of the sample during mechanical experiments (Additional File 2: Fig. S6E–H). This capability allows detailed observation of sample behavior under mechanical stress. When used with an inverted microscope, the Camelot system maintains its adaptability. As shown in Additional File 2: Fig. S7, the baseplate is securely mounted on adjustable brackets, aligning the force sensor and other components with the optical path (Additional File 2: Fig. S7 A–C). Samples positioned within a Petri dish are located for simultaneous imaging and force measurements, while the modular design ensures accessibility and alignment of all components (Additional File 2: Fig. S7D–H).

### Control software

The Camelot system is controlled by MorphoRobotX (www.MorphoRobotX.org) (Fig. 2), which serves as the control software for the Cellular Force Microscope [22, 32], and various extensometer setups [15, 21, 23]. The graphical user interface for MorphoRobotX is modeled after MorphoGraphX [28], and users are encouraged to familiarize themselves with MorphoGraphX to better understand its layout and functionality. As with MorphoGraphX, MorphoRobotX organizes tasks into processes, which manage key operations such as stage movement, sample stretching, calibration, and parameter settings. These processes also represent hardware components (drivers), including the camera, actuator, and force sensor, and come pre-configured with experimental defaults. Additional hardware elements (actuators, cameras, acquisition devices) can be added via a plug-in system that allows the incorporation of additional processes. Throughout each session, MorphoRobotX creates logs, recording data such as forces, stage positions, and camera images which are synchronized with extensometer steps as the experiment progresses.

### Incorporating new hardware

MorphoRobotX allows the incorporation of additional force sensors, actuators, and cameras through shared libraries that contain hardware processes. Although the Phidgets bridge amplifier is compatible with most resistive strain gauge sensors, processes are also available for Futek USB amplifiers as well as for analog amplifiers using various data acquisition cards via the Linux Comedi library (for example, the National Instruments PCI- 6321). Actuators currently supported include Phidgets and Zaber stepper motor-driven screw drives, as well as SmarAct and SmarAct2 piezo-driven actuators. MorphoRobotX can use any camera supported by OpenCV, although this may require installing vendor-supplied libraries. Camera drivers are also available for IDS and PVCam. A process is provided to enable the use of a selection of Micro-Manager-supported cameras, such as DCAM/IIDC-standard Firewire cameras. For devices not covered above, it is possible to write new processes in C++ by inheriting from base classes to call vendor-supplied libraries directly. These can then be compiled as shared libraries, placed in the MorphoRobotX process directory, and loaded on startup.

### Experimental workflow

The experimental workflow for the Camelot system covers the key steps required for conducting mechanical testing and imaging experiments. It begins with calibrating the system hardware to achieve reliable measurements, followed by preparing the samples with appropriate mounting and hydration methods. Finally, the samples are stretched using either manual or automated procedures, allowing for accurate force application and imaging. This workflow is adaptable to various experimental setups and research objectives, providing flexibility while maintaining consistency in data collection.

### System calibration

To achieve accurate force measurements with the load cell, the sensor’s gain must be calibrated to accurately convert voltage readings into force. We encourage users to do this periodically, and to verify the calibration before and after experiments to ensure the sensor is not damaged.

#### Measure and calculate the weight of a known load

Use an analytical balance to measure the weight of the chosen calibration object (e.g., a screw, nut, or bolt) multiple times to minimize variability. Calculate the average weight by summing all measurements and dividing by the number of measurements. Convert the weight into force using the formula $F=m\times g$, where $g=9.81m/{s}^{2}$, with 1 g equivalent to 9.806 mN. Record this theoretical force as your reference value. For calibration, choose weights that match the expected force range of your sample. In our standard measurements with etiolated Arabidopsis hypocotyls, we used a 5 g calibration weight (approximately 49.1 mN) with a 10 g load cell, which is well-suited for these soft tissues. For stiffer tissues, such as mature stems or thicker leaves where higher forces are expected, a heavier calibration weight such as 10 g (approximately 98.1 mN) or 20 g (approximately 196.2 mN) is recommended. In these cases, a load cell with a larger capacity, for example a 100 g load cell, should be used to match the increased dynamic range.

#### Verify hardware defaults and initialize components

Before proceeding, confirm that the defaults for the sensor, actuator, and camera are correctly configured under “Tools/MorphoRobotX/Experiment Defaults.” Begin by initializing the actuator through “Tools/MorphoRobotX/Actuator/Phidgets Positioner.” Then, initialize the sensor by double-clicking on “Tools/MorphoRobotX/Sensors/Phidgets Sensor” to confirm that MorphoRobotX can communicate effectively with the hardware. If successful, nothing will happen, but if not, an error box will pop up.

#### Prepare the sensor and set the offset

Once the hardware is properly initialized, move Camelot and the load cell to a vertical position using the 3D-printed calibration stand or any stable L-bracket to securely support the load cell. Open “Tools/MorphoRobotX/Sensors/Set Offset” and run the Set Offset process with the sensor alone, ensuring no weight is on the load cell. This action zeroes the sensor, removing any residual force readings. Then navigate to “Tools/MorphoRobotX/Experiment/Monitor Force,” activate the Monitor Force process to display real-time force measurements and confirm that the displayed force values are around zero.

#### Add the weight and check the force

Place the known weight on the load cell. Use “Tools/MorphoRobotX/Experiment/Monitor Force” to observe the force value measured by the sensor. Use the “Tools/MorphoRobotX/Sensors/Calibrate Force” process with the previously calculated value for the reference weight in the “Target Force” parameter. This will calculate the correct sensor gain and write it to the “Sensor Gain” parameter of the force sensor process.

#### Mitigate environmental noise

If fluctuations are observed in the force readings during calibration or use, consider addressing potential environmental factors. Noise can result from temperature changes, vibrations, or electromagnetic interference. To minimize these effects, ground the experimental setup and, if necessary, place the load cell and related components inside an isolation box. Additionally, use a vibration isolation table with pneumatic supports, which helps dampen external mechanical vibrations. These measures stabilize the sensor’s performance and ensure the reliability of its readings.

#### Validate the calibration and document

After calibration, remove the weight from the load cell and return Camelot to a horizontal position. Use “Tools/MorphoRobotX/Experiment/Monitor Force” to check the force. Since the weight of the sensor will affect the force, rerun “Tools/MorphoRobotX/Sensors/Set Offset” to zero it in the horizontal position. Document the final gain value (from the “Sensor Gain” parameter on the sensor process) for future reference. A large change in this value could indicate damage to the sensor. The sensor is now ready for precise force quantification in experimental applications.

### Sample preparation

Sample preparation for experiments using the Camelot system involves preparing adhesive tags as mounting points for tissue samples, selecting or dissecting samples according to the experimental design, and attaching the tissue ends to the tags. Artificial landmarks can be added to the samples to track deformation during testing. Hydration is maintained throughout to minimize changes in tissue properties. The prepared samples are mounted on the Camelot setup and aligned appropriately for stretching, with attention to uniform tension and proper positioning.

#### Prepare the adhesive tags

Before starting the experiment, prepare adhesive tags such as Tough-Tags (Diversified Biotech, Cat. No. TTLC- 1000) or NIIMBOT Thermal Transparent Stickers. Punch holes in the tags using a hole puncher, making sure the holes are appropriately sized and positioned for mounting on the Camelot setup pins. If needed, apply an adhesive scale bar directly onto the tags, especially for small samples. Alternatively, if the adhesive tape has a known dimension (e.g., the Tough-Tags width of 12.7 mm), this can serve as a built-in scale bar.

#### Select or dissect the tissue

Once the adhesive tags are prepared, select or dissect the tissue sample according to the experimental requirements. For small samples, such as Arabidopsis hypocotyls or epidermal peels, use the prepared adhesive tags for mounting. For larger or thicker samples, such as pine or elm hypocotyls, consider using stronger adhesion methods, such as gluing the sample ends into small rubber tubes that can be secured with clamps [33].

#### Mount the tissue ends onto adhesive tags

Attach the ends of the tissue sample to the prepared adhesive tags. Fold each tag in half over the tissue to ensure full adhesion and even distribution of tensile force. Press the tags firmly to prevent slippage during the experiment. Verify that the tissue is aligned centrally within the tags for consistent stretching.

#### Apply landmarks

To track tissue deformation, put landmarks on the tissue using a very thin, soft, waterproof marker, such as a fine eyeliner or permanent marker. These landmarks will assist in measuring changes in length and distance during stretching. Make sure that the application of the markers does not damage or deform the tissue (Fig. 2C). To obtain representative Young’s modulus for the whole tissue, landmarks should be placed as far apart as possible within regions of similar cross-sectional area. This minimizes noise from small-scale variations in strain. If measuring stiffness gradients, such as along the length of a hypocotyl, landmarks should be positioned at multiple points along the growth axis to capture spatial differences. For higher-resolution measurements, a microscope with a camera can be used to track deformation at the cellular level by following cell landmarks instead of manually placed landmarks.

#### Prevent dehydration

To prevent dehydration-related changes in tissue properties, place the prepared sample in water temporarily while additional samples are being prepared. Alternatively, if the sample is to be stretched immediately, proceed with mounting and stretching promptly to minimize dehydration.

#### Mount the sample on the Camelot setup

Transfer the prepared sample to the Camelot setup. Depending on the preparation method, either float the sample on the water surface of a water-filled plate before mounting or directly mount it onto the pins if stretching immediately without additional hydration. Verify that the adhesive tags are positioned correctly for secure attachment.

#### Secure the sample on the pins

Use forceps to handle the sample and carefully position the adhesive tags onto the pins or bolts of the Camelot setup. Push the tags firmly down onto the pins to confirm they are securely mounted. If the sample is in a water-filled plate, confirm that it is fully submerged and stabilized for the stretching process.

#### Align the sample for stretching

Adjust the actuator of the Camelot setup using “Tools/MorphoRobotX/Actuators/Move Actuator” to align the sample properly. Check whether the tissue is straight to help spread the tension evenly. Verify that the adhesive tags are securely mounted on the pins and that the sample is free from twisting or bending. Once the alignment is complete, the sample is now ready to be stretched.

### Stretching

The system can be operated either manually or automatically. Manual operation is often used to evaluate system behavior and determine key parameters, such as the required step size and the stabilization time between steps. Automatic operation enables precise stretching, allowing the system to record each step across the range of applied forces while synchronizing images captured by the camera or microscope.

### Manual stretching

#### Capture an initial image

Capture an image of the sample in its relaxed state. Ensure that the sample is well-aligned, and the scale bar and any landmarks are visible in the image.

#### Stretch the sample

Stretch the sample incrementally by moving the actuator using “Tools/MorphoRobotX/Actuators/Move Actuator.” Enter the desired distance for each stretch in the “Move Measure” parameter. Monitor the resulting force after each stretch using “Tools/MorphoRobotX/Experiment/Monitor Force” for consistent application of tension.

#### Allow the force to stabilize

After each actuator movement, allow the force to stabilize before further stretching.

#### Capture images at each stretching point

If a camera is integrated, open the camera interface through “Tools/MorphoRobotX/Camera/OpenCV Camera.” Use the “Take Snapshot” button in the camera window to capture images at each stretching point, ensuring documentation of the sample’s deformation throughout the experiment.

### Automated stretching

#### Activate the camera

Open the camera interface by double-clicking the appropriate camera type in “Tools/MorphoRobotX/Camera” folder. Check whether the camera feed is active, and the sample is in focus to capture the sample’s deformation throughout the experiment.

#### Configure automated stretching

Initiate the automated stretching process by opening “Tools/MorphoRobotX/Experiment/Extensometer.” Set the total distance to be covered during the experiment using the “Distance” parameter. Define the step size with the “Step Size” parameter. Adjust step sizes based on sample characteristics. Smaller step sizes yield more data but increase experiment duration, while larger step sizes may risk sample damage.

#### Configure the wait time

If required, adjust the “Wait Time” parameter to change the amount of time to wait for force stabilization between steps. If the system is being used with a confocal microscope or a camera that is not integrated with MorphoRobotX, set this time to − 1 and the system will pop-up a window and wait for confirmation that the image has been captured before proceeding to the next step.

#### Creep experiments

For creep experiments, specify the starting force threshold using the “Creep Threshold” parameter. The system will stretch the sample at a constant rate until the specified force is reached, then make steps periodically as required to maintain that force. For elasticity experiments, the “Creep Threshold” to 0, which is the default.

#### Start the stretching experiment

Begin the experiment by double-clicking “Tools/MorphoRobotX/Experiment/Extensometer.” Real-time force readings and a live camera feed will display, and snapshots of the stretching sample will be saved automatically to disk.

#### Return to the starting position

Once the total distance set for the experiment is reached, the actuator will automatically return to its starting position, completing the stretching cycle.

#### Cancel if necessary

If it is required to interrupt the experiment to stop the stretching, press the “Stop” button in the upper right-hand side of the MorphoRobotX window. Force readings and snapshots of the stretching sample will be saved automatically to disk. Use “Tools/MorphoRobotX/Actuators/Move Actuator” to manually return the actuator to its starting position and reset the setup as needed.

### Data analysis

Each automated extensometer experiment produces three sets of files, an Extensometer*.csv* file, a Snapshots folder with camera images for each step in the experiment, and an MRXlog*.*csv file containing raw data from the sensor and actuator. These are named with a date and time stamp of when the experiment started. The Extensometer file has columns Position (nm), Force (µN), and Time (µs) data for each step of the extensometer experiment. Each actuator step is associated with a snapshot and a corresponding force value. For manual stretching, data is manually collected from both the relaxed and stretched states. Landmarks from snapshots or confocal scans, such as cell junctions, are used to measure linear distance changes. Confocal data segmented using MorphoGraphX (MGX) can provide information on area or volume changes. A force curve can be generated by plotting actuator steps against force. To determine stress, two points within the elastic region of the force curve are selected, and their corresponding actuator steps are analyzed. Snapshots from these steps are used to measure landmark displacements, such as cell junctions or applied markers, which are then used to calculate strain.

Mechanical properties are assessed by calculating stress and strain from force–displacement data recorded during the extensometer experiment. Stress ($\sigma$) is calculated by dividing the applied force ($F$) by the sample’s cross-sectional area ($A$). For cylindrical samples, such as hypocotyls, stems, or roots, the cross-sectional area is determined using the formula $A=\pi {r}^{2}$, where $r$ is the radius. The radius can either be calculated by measuring the diameter of the sample under tension with the integrated digital microscope camera and halving it or by preparing cross-sections and averaging the radius of samples of the same genotype. For non-cylindrical samples, such as leaves, sepals, or epidermal peels, cross-sectional areas require different approaches. Cross-sections can be obtained to calculate an average area for the specific sample type. Alternatively, for approximate calculations, the known or measured thickness of the sample can be combined with its width to estimate the cross-sectional area as $A=thickness\times width$. Strain ($\varepsilon$), expressed as a percentage, represents the relative elongation of the sample and is calculated as $\varepsilon =\frac{\Delta L}{{L}_{0}}\times 100$. Here, $\Delta L$ is the change in length, determined as the difference between the stretched length just before rupture and the initial length under tension (${L}_{0}$) [34].

Analysis typically focuses on the linear region of the stress–strain curve, where deformation is proportional to the applied force, following Hooke’s Law ($\sigma =E\times \varepsilon$), with $E$ as the elastic modulus [34]. This part of the curve typically avoids plastic deformations, capturing reversible elastic deformations where the sample returns to its original shape upon force removal. Strains are typically limited to below 10–20% so that they remain within the range that would normally be experienced by the plant cell wall. These can be much higher when measuring failure stress or plasticity. By isolating the linear region, a single number for the elastic modulus can be calculated for each sample.

### Parts list

Tables 1 and 2 show the parts list.

## Supplementary Information

Additional file 1. Table S1. Comparison of microextensometer systems for plant biomechanics. This table compares actuation, force sensing, deformation tracking, software integration, and cost across recent extensometer setups. The Camelot system (Trozzi et al., 2025) is presented alongside ACME (Robinson et al., 2017), Bidhendi et al. (2020), Lee et al. (2024), Chen et al. (2024), and Hofhuis et al. (2016), showing differences in precision, complexity, and affordability.

Additional file 2. Figures S1–S7. Fig. S1 – Hardware components. Fig. S2 – 3D-printed parts. Fig. S3 – Setup configurations. Fig. S4 – Electronic layouts. Fig. S5 – Confocal setup. Fig. S6 – Inverted microscope setup. Fig. S7 – Confocal stretch series.

Additional file 3. Dataset: Files for 3D-printed components and Debian package for MorphoRobotX. The zip file contains all the necessary files for 3D-printing the Camelot components and the Debian package for MorphoRobotX.

Additional file 4. Movie 1. Time-lapse video of digital microscope images during stretching. A video composed of sequential images captured during the stretching experiment of Arabidopsis xxt1 xxt2 hypocotyl.

Additional file 5. Movie 2: Time-lapse video of confocal images during stretching. A video composed of sequential confocal images captured during the stretching experiment, showing the progression from 0% to 9.5% strain. Displacement data are annotated throughout the video. Scale bar: 50 µm.

## Data Availability

No datasets were generated or analysed during the current study.

## Abbreviations

ACME: Automated confocal micro-extensometer
CCD: Charge-Coupled Device
CFM: Cellular force microscope
CH: Switzerland (Confoederatio Helvetica)
CNC: Computer Numerical Control
DCAM: DCAM-compatible digital camera standard
DIY: Do-It-Yourself
GBP: British Pound Sterling
IDS: Imaging Development Systems
IIDC: Industrial Instrumentation Digital Camera standard
IQR: Interquartile range
LSM: Laser scanning microscope
MGX: MorphoGraphX
NEMA: National Electrical Manufacturers Association
NIIMBOT: Commercial brand of thermal label printers
NLO: Nonlinear optics
PCI: Peripheral component interconnect
TTLC: Tough-Tags Label Code
UK: United Kingdom
USB: Universal Serial Bus
VINT: Versatile Interface for Next-generation Technologies
YFP: Yellow Fluorescent Protein
