# Supplementary material for: Camelot: a computer-automated micro-extensometer with low-cost optical tracking
Source: BMC Biol. 2025 Apr 28;23:112. doi: 10.1186/s12915-025-02216-9 (PMC12036183; doi:10.1186/s12915-025-02216-9)
Supplement: Supplementary file 1 — Additional file 1. Table S1. Comparison of microextensometer systems for plant biomechanics. This table compares actuation, force sensing, deformation tracking, software integration, and cost across recent extensometer setups. The Camelot system (Trozzi et al., 2025) is presented alongside ACME (Robinson et al., 2017), Bidhendi et al. (2020), Lee et al. (2024), Chen et al. (2024), and Hofhuis et al. (2016), showing differences in precision, complexity, and affordability. [file 12915_2025_2216_MOESM1_ESM.docx]

**Additional File 1: Table 1: Comparison of microextensometer systems for plant biomechanics.** This table compares actuation, force sensing, deformation tracking, software integration, and cost across recent extensometer setups. The Camelot system (Trozzi et al., 2025) is presented alongside ACME (Robinson et al., 2017), Bidhendi et al. (2020), Lee et al. (2024), Chen et al. (2024), and Hofhuis et al. (2016), showing differences in precision, complexity, and affordability.

| **System (reference)** | **Actuation technology** | **Force sensing** | **Deformation sensing** | **Software & integration** | **Estimated cost** |
| --- | --- | --- | --- | --- | --- |
| **Camelot** (Trozzi et al., 2025) | Low-cost stepper motor driving a lead screw on a linear rail to stretch the sample​. This screw-drive actuator provides ~5 µm displacement resolution per step. | Employs a small single-point load cell (parallel-beam strain gauge type, like those in digital scales) mounted as the stationary clamp​. A 10 g capacity load cell gives ~10 µN force accuracy​. The load cell output is amplified via a Phidgets Wheatstone bridge interface for computer reading​​. | Relies on optical tracking of sample deformation using a camera. A compact digital microscope or C-mount microscope camera tracks marked points on the sample during stretching​ . This image-based measurement overcomes slip or alignment errors in purely actuator-based readings. Camelot can also be placed on microscopes (including confocal) to use cell details as natural fiducial markers​. | Controlled by MorphoRobotX, an open-source software that integrates actuator control, force acquisition, and imaging. The software runs on Linux and has been verified to work on Windows using Windows Subsystem for Linux (WSL). It provides a unified control interface that synchronizes motor steps with image capture and force logging. The system is modular and portable, supporting a variety of actuators, force sensors, and cameras, making it adaptable for high-resolution optical tracking on different microscope setups. | **Rough total: ~500 GBP** • Actuator and control electronics (stepper motor with screw-drive linear stage, motor controller, and associated wiring and power supply): ~150 GBP • Force sensor module (10 g load cell, Wheatstone Bridge, VINT Hub, and necessary cabling): ~75 GBP • Optical tracking unit (compact digital microscope camera): ~100 GBP • Integration and mounting hardware (2‑axis manual stage, 3D‑printed components, nuts, bolts, and miscellaneous small parts): ~175 GBP |
| **ACME** (Robinson et al., 2017) | Two high-precision SmarAct piezoelectric linear nano-positioners (model SLC-1720) pull the sample. These stick-slip piezo stages offer nanometer-scale resolution and a ~17–20 mm travel range. One moving arm actuator applies displacement, while a second measuring arm adjusts position on the opposite side. The SmarAct stages are controlled via a SmarAct MCS-3D controller. | A miniature Futek LSB200 load cell (10 g capacity) mounted at the measuring arm records force. Its signal is amplified (Futek CSG110 amplifier) and acquired through a National Instruments DAQ (NI PCI-6221 with SCB68 breakout). This setup provides high-resolution force data in the millinewton to micronewton range. | Confocal microscopy is used to measure tissue deformation at cellular resolution. Instead of relying solely on-stage motion, ACME captures confocal image stacks of the sample during stretching and computes strain from biological landmarks (e.g., cell wall fluorescent markers) in 3D. This allows mapping heterogeneous deformation within the tissue. | Custom software controls the SmarAct actuators and force sensor but does not directly trigger image capture from the confocal microscope. Instead, confocal imaging must be manually synchronized with mechanical testing. | **Rough total: ~10,600 GBP** • SmarAct nano-positioning system (two SLC-1720 stages with MCS-3D controller): ~8,000 GBP • Miniature load cell and signal conditioning (Futek LSB200, CSG110 amplifier, NI PCI-6221 DAQ with SCB68 breakout): 2,500 GBP • Integration hardware and mounting components: ~100 GBP |
| **Bidhendi *et al.*, 2020** | Motorized linear translation stage (Thorlabs MTS25/M-Z8 DC servo stage)​. This stage offers a 25 mm travel range and fine control (minimum 0.8 µm step size, <6 µm backlash)​. It is driven by a Thorlabs KDC101 controller, allowing programmed speed (up to 2.4 mm/s) and position control via a PC​ | Small single-point shear beam load cell (Phidgets CZL639HD) with 100 g capacity​. This strain-gauge load cell is read by a Phidgets Wheatstone Bridge board, providing digital force data via USB. With 5 V excitation, the load cell yields ~3 mV at full load, and the system achieves roughly 0.5 g (~5 mN) force resolution in practice​. The setup can swap in different load cells (10 g to 5 kg range) as needed​​, | Features an independent displacement sensor in the form of a DC LVDT (Linear Variable Differential Transformer) attached to the moving grip​. The LVDT (Trans-Tek model, ±76 mm range) provides contactless measurement of the extension with theoretically infinite resolution​. By measuring actual grip movement, it corrects for any stage backlash or compliance, ensuring accurate strain data​. (The motor’s own encoder steps might not equal real sample deformation due to slippage or delays, so the LVDT feeds true displacement feedback​). | The stage is operated through Thorlabs APT software (or Kinesis), where speed, acceleration, and motion profiles can be set for tensile tests​. Sensor data (force from Phidgets bridge and displacement from LVDT) are acquired with a custom Python script using the Phidgets API​. The script plots force-displacement in real time and saves data to CSV​. This modular design is mounted on a rigid base and can be used under a stereomicroscope or adapted for confocal by using smaller components​. | **Rough total: ~2,300 GBP** • Motorized linear translation stage and controller (Thorlabs MTS25/M-Z8 with KDC101): ~1,800 GBP • Single-point shear beam load cell and digital interface (Phidgets CZL639HD with Wheatstone Bridge board): ~75GBP • Independent DC LVDT displacement sensor (Trans-Tek model): ~300 GBP • Integration hardware and mounting components: ~100 GBP |
| **Lee *et al.*, 2024** | Miniature New Scale Technologies M3-LS linear micro-stage to apply tensile strain​. This piezoelectric motor-driven stage has an internal screw mechanism and 0.5 µm resolution per step​. It provides precise electronic control of displacement via New Scale Pathway software​. The compact stage was chosen to fit onto a specialized microscopy setup (SFG imaging system)​ | Futek LSB205 miniature load cell attached to one clamp​. A glass slide is mounted on the load cell to hold the sample flat, and the opposing end of the sample is affixed to the movable stage extension​. Force readings are digitized through a Futek USB220 amplifier/converter and recorded with Futek SENSIT software on a PC​. This setup measures forces while the sample is stretched, likely in the μN to mN range, suitable for thin onion epidermis strips. | Primarily uses the commanded stage displacement for strain, since the stage has high positional accuracy​. The system was designed to minimize slippage, a custom I-block/Z-block clamping mechanism holds the onion epidermis without glue, ensuring that stage motion translates to sample stretch​. During tests, samples were stretched at a constant speed (200 µm/s, ~0.01 s^−1^ strain rate) and then imaged after ~50 s to observe any residual strain​. (Optical imaging was used only before/after to track cell positions, not continuously during stretch​.) | New Scale Pathway software controlled the M3-LS stage motion (allowing preset velocity and displacement)​. Force data collection is handled separately by Futek SENSIT program via the USB load cell interface​. The two systems (motion and force) are likely started in sync manually. The entire extensometer was built to integrate with a Sum Frequency Generation (SFG) microscope, meaning it had a very low profile and precise alignment to fit under objective lenses​. | **Rough total: ~5,000 GBP** • New Scale Technologies M3-LS linear micro‐stage with Pathway controller: ~3,000 GBP • Futek LSB205 miniature load cell with USB220 amplifier/converter (including SENSIT software): ~1,300 GBP • Custom integration components (3D‐printed stage extension, I-block/Z-block clamping system, glass slide, wiring, etc.): ~700 GBP |
| **Chen *et al.*, 2024** | Uses a Newport AG-LS25 piezoelectric linear stage, mounted on a Newport MS-125-XYZ manual stage, to apply tensile strain at a constant velocity (~2.5 µm/s) with sub-micron displacement resolution. | Incorporates a Futek LSB200 miniature load cell for continuous force monitoring during stretching. | Measures deformation optically via confocal microscopy. Fluorescent beads deposited on the sample surface serve as fiducial markers, and a Zeiss LSM710 upright confocal microscope captures time-series images to compute strain with cellular resolution. | Custom control software synchronizes piezo stage movement, force data acquisition from the load cell, and confocal image capture. This integrated approach allows real-time correlation of mechanical loading and optical measurement without requiring separate platforms for each component. | **Rough total: ~5,765 GBP** • Piezo linear stage and controller: ~1,120 GBP (AG-LS25: ~720 GBP + controller: ~400 GBP) • Manual stage (MS-125-XYZ): ~645 GBP • Miniature load cell and signal conditioning electronics: ~2,000 GBP • Integration hardware and data acquisition components: ~2,000 GBP |
| **Hofhuis et al., 2016** | A piezoelectric micropositioner arm (SLC‑2475 from SmarAct GmbH) that moves in discrete 50‑µm increments. | A miniature load cell (Futek LSB200) mounted on one clamp, which records the tensile force via its strain‐gauge signal. | The displacement is set by the piezo stage 50‑µm steps; additional qualitative imaging is provided by a high‑magnification (300×) webcam to verify valve flattening. | Custom control is achieved through scripts written in Octave that coordinate the piezo stage movement with force data acquisition from the load cell. The system is built from bespoke components with simple integration (no full commercial platform). | **Rough total: ~7,500 GBP** • Piezo actuator (SLC‑2475): ~6,000 GBP  • Load cell (Futek LSB200): ~1,000 GBP • High‑magnification webcam: ~150 GBP • Data acquisition, wiring & electronics: ~300 GBP • Miscellaneous fabrication & hardware: ~250 GBP |
