# Supplementary material for: Camelot: a computer-automated micro-extensometer with low-cost optical tracking
Source: BMC Biol. 2025 Apr 28;23:112. doi: 10.1186/s12915-025-02216-9 (PMC12036183; doi:10.1186/s12915-025-02216-9)
Supplement: Supplementary file 2 — Additional file 2. Figures S1–S7. Fig. S1 – Hardware components. Fig. S2 – 3D-printed parts. Fig. S3 – Setup configurations. Fig. S4 – Electronic layouts. Fig. S5 – Confocal setup. Fig. S6 – Inverted microscope setup. Fig. S7 – Confocal stretch series. [file 12915_2025_2216_MOESM2_ESM.docx]

**Additional File 2**

**Camelot: a Computer Automated Micro Extensometer with Low-cost Optical Tracking**

Nicola Trozzi^+,1,2, 3^, Wiktoria Wodniok^+,4^, Robert Kelly-Bellow^+,1^, Andrea Meraviglia^2^, Aurore Chételat^2^, Nova Adkins^1,3^, Brendan Lane^1^, Richard S. Smith^✉,1^, Dorota Kwiatkowska^✉,4^, Mateusz, Majda^✉,2^

^1^ Department of Computational and Systems Biology, John Innes Centre, Norwich NR4 7UH, UK

^2^ Department of Plant Molecular Biology, University of Lausanne, CH-1015 Lausanne, Switzerland

^3^ School of Biological Sciences, University of East Anglia, Norwich, NR4 7TJ UK

^4^ Institute of Biology, Biotechnology and Environmental Protection, Faculty of Natural Sciences, University of Silesia, 40-032 Katowice, Poland

^+^ These authors contributed equally.

✉To whom correspondence may be addressed: Mateusz Majda (Mateusz.Majda@unil.ch), Dorota Kwiatkowska (dorota.kwiatkowska@us.edu.pl), Richard S. Smith (Richard.Smith@jic.ac.uk).


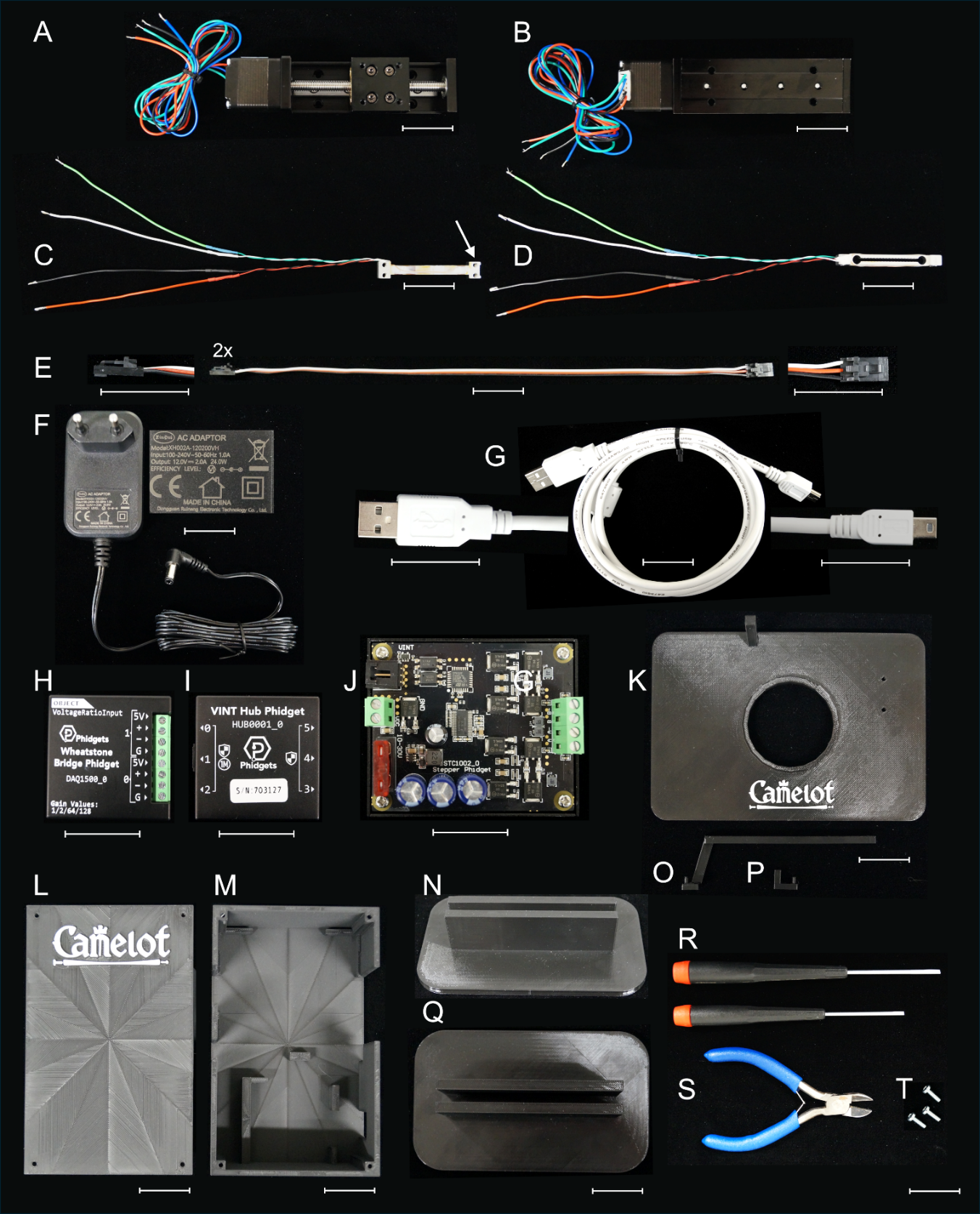


**Additional File 2: Fig. S1: Components of the experimental setup.** (**A**) Linear motion stage with stepper motor (THK KR20 Linear Stage), showing the rail system and mounting plate. The bundled wires connect the stepper motor to the controller. (**B**) Side view of the linear motion stage. (**C**) Close-up of the strain gauge sensor (Phidgets 3133_0 Micro Load Cell) connected to the wiring harness, with an arrow indicating the strain-sensitive region of the sensor. (**D**) Load cell sensor (Phidgets 3134_0), featuring a different design and mounted on a custom attachment for measuring higher force ranges. (**E**) Connector cables (Phidgets Interface Kit 8/8/8) with standard plugs for signal transmission. (**F**) AC power adapter (Mean Well GST25A05-P1J) supplying regulated 5V power for system operation. (**G**) USB cable (Phidgets USB Cable 3002_0) for transmitting data between the setup and the computer for real-time monitoring and control. (**H**) Wheatstone Bridge Phidget module (DAQ1500) for converting low-voltage analog signals from the strain gauge into digital data for analysis. (**I**) VINT Hub Phidget (HUB0001), providing a central interface to connect multiple devices via USB. (**J**) Motor controller board (Pololu Tic T825 USB Multi-Interface Stepper Motor Controller), used for motion control of the linear stage. (**K**) Custom 3D-printed mounting plate with a central hole for cable routing and alignment of components. (**L**) External view of the 3D-printed housing designed to protect internal components from environmental damage. (**M**) Internal view of the 3D-printed housing, showing compartmentalized layouts to accommodate the motor controller, hub, and wiring, with open sides for ventilation. (**N**) Additional 3D-printed structural supports designed for reinforcing sensor attachments and minimizing vibrations during operation. (**O**) Custom-printed alignment attachment for positioning of the strain gauge sensor during calibration. (**P**) Secondary alignment component to stabilize the load cell sensor, ensuring consistent measurements. (**Q**) Structural insert for securing internal components and maintaining system rigidity. (**R**) Screwdrivers (Wera 334/6 Screwdriver Set) used for assembly, including both flathead and Phillips head types for various screw sizes. (**S**) Wire cutters (Knipex 70 06 160) for trimming and preparing cables during installation and maintenance. (**T**) Screws and bolts (M3-M6 stainless steel fasteners, sourced from McMaster-Carr) for securely attaching components to the mounting plate and housing. Scale bars: 30 mm.


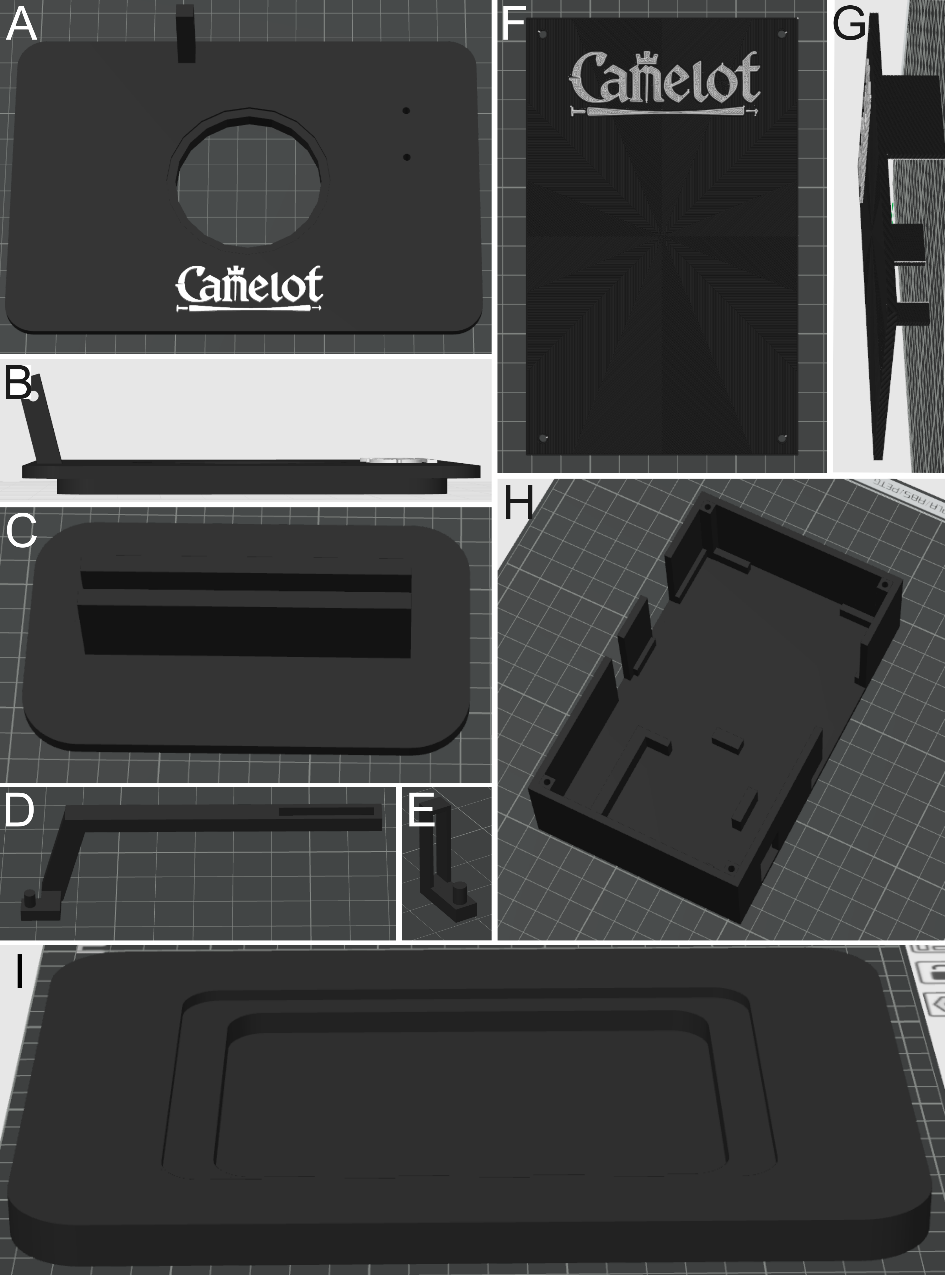


**Additional File 2: Fig. S2: 3D-printed components of the Camelot setup.** (**A**) Top view of the microscopy stage, including a central hole with a small indentation for positioning a small Petri plate. Two additional holes on the right side are designed for mounting the actuator, while the pole near the central hole provides a mounting point for the sensor. (**B**) Side view of the microscopy stage fitting seamlessly onto confocal, two-photon, or epifluorescence microscope setups. (**C**) Calibration stage with a central gap to securely hold the microscopy stage during calibration procedures. (**D**) Arm for the micro-extensometer connected to the actuator. The arm features a pin for mounting samples with adhesive tape, such as Tough-Tags. (**E**) Arm for the micro-extensometer connected to the sensor, equipped with a pin for attaching the opposite side of the sample. (**F**) Top view of the lid of the electronics box, designed to house and secure the stepper controller and the hub. Four corner holes allow the lid to be mounted onto the box bottom part. (**G**) Side view of the electronics box lid, showing side extensions that cover wiring openings for additional protection. (**H**) Top view of the electronics box bottom part, including holders for the stepper controller on the top section and the hub on the bottom section. Missing side walls accommodate wiring, while four corner holes allow secure mounting of the lid to enclose the box and protect the components. (**I**) Holder for the microscopy stage when used without a microscope.


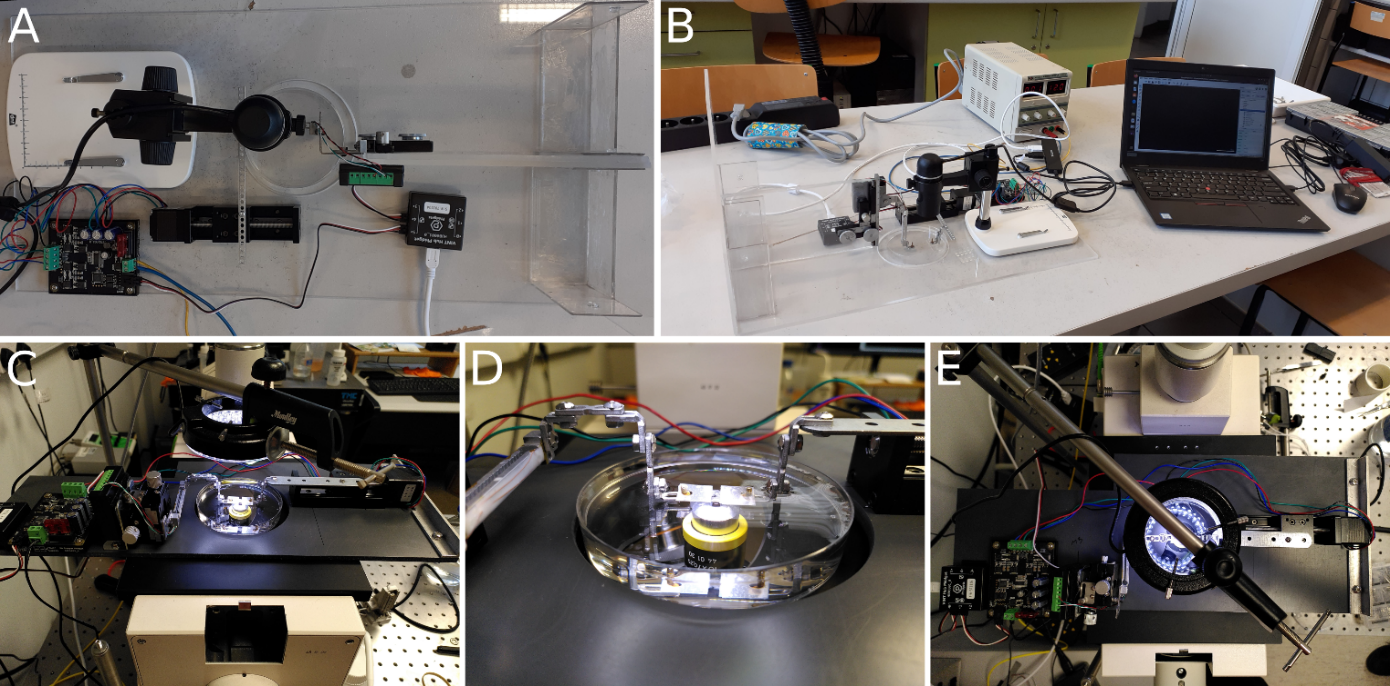


**Additional File 2: Fig. S3: Alternative Camelot setup configurations for mechanical measurements and imaging.** The Camelot system is depicted in two configurations for different experimental setups. Basic Camelot setup for use with a digital microscope: (**A**) The standalone Camelot system showing the force sensor, actuator, and sample holder, integrated with a compact USB digital microscope. The system is enclosed in a transparent housing for environmental protection, with electronic components, including the Phidgets Wheatstone Bridge and motor controller, visibly connected. (**B**) A broader view of the basic setup showing the connections to a laptop for real-time data acquisition and control. The modular design facilitates easy integration with consumer-grade digital microscopes for capturing deformation data. Camelot setup configured for use with an inverted microscope: (**C**) Overview of the Camelot system mounted on an inverted microscope stage. (**D**) Close-up view of the sample mounted on the force sensor under a Petri dish. The modular sample holder is shown in detail. (**E**) Top view of the setup on the inverted microscope stage.


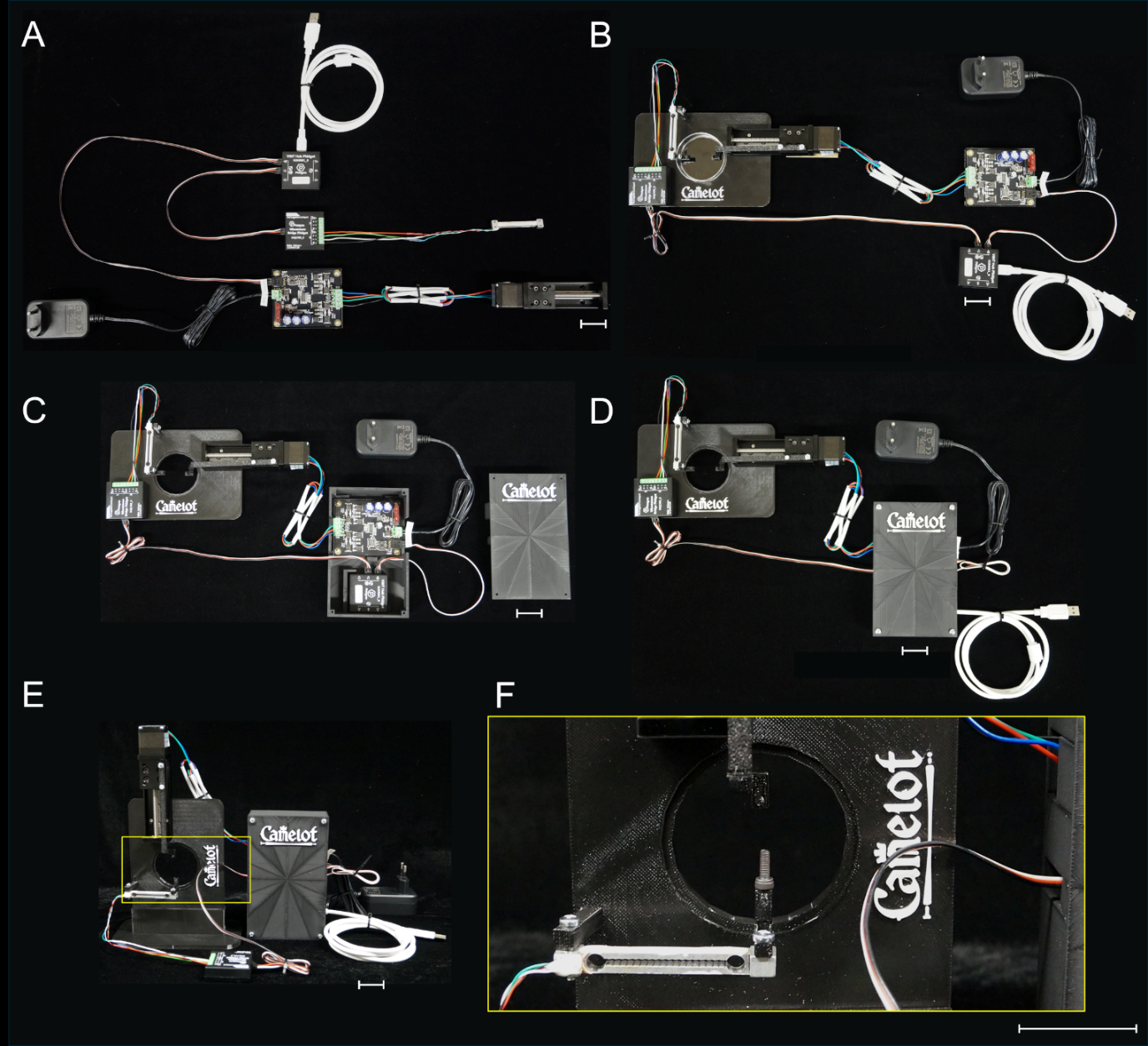


**Additional File 2: Fig. S4: Configurations of the experimental setup.** (**A**) Baseline configuration: The setup shows the core connections, with the strain gauge sensor directly mounted on the linear motion stage and connected to the Wheatstone Bridge Phidget, which in turn is linked to the VINT Hub. The VINT Hub connects to the computer via USB for data acquisition. The motor controller drives the stepper motor, powered by an AC adapter. (**B**) Printed stage configuration: This setup builds on (A) by mounting the components, including the linear motion stage and strain gauge sensor, onto a custom 3D-printed "Camelot" baseplate. (**C**) Enclosed configuration: A 3D-printed housing is added to protect the motor controller and VINT Hub. The housing organizes the wiring and shields electronic components from environmental factors while maintaining the horizontal orientation of the strain gauge sensor. (**D**) Enclosed configuration with extended housing: Similar to (C), but with a larger 3D-printed housing that fully encloses the electronic components and provides additional structural protection. This configuration improves compactness and organization for experiments involving horizontal force measurements. (**E**) Vertical calibration configuration: The setup is reconfigured to position the strain gauge sensor vertically on the printed baseplate. This orientation allows for calibration of the sensor for force measurements. (**F**) Magnified view of the vertical configuration: Close-up of the strain gauge sensor and its custom 3D-printed holder. The connection between the strain gauge and the Wheatstone Bridge Phidget is shown. Scale bars: 30 mm.


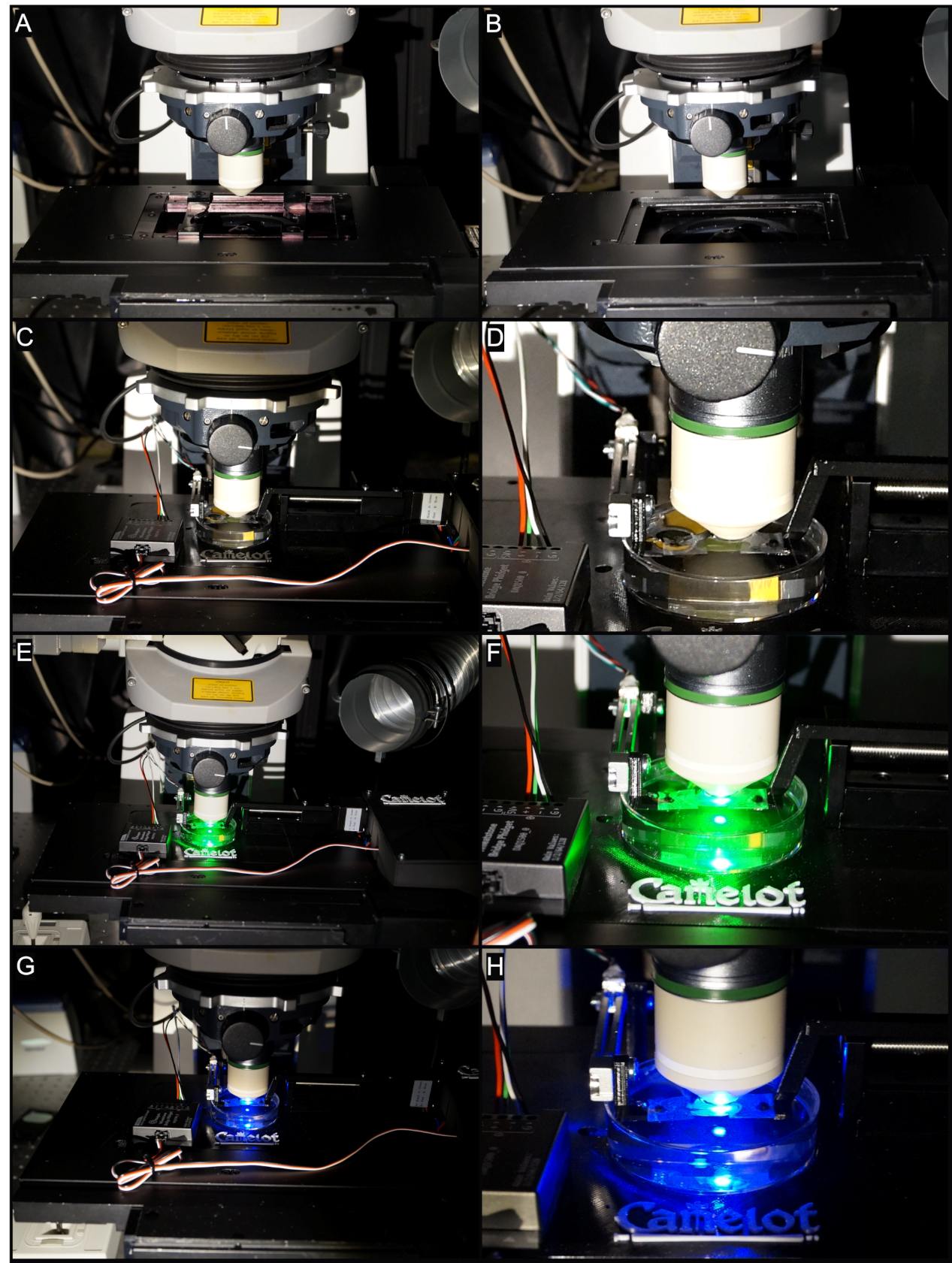


**Additional File 2: Fig. S5: Operation of the experimental setup under a confocal microscope for simultaneous mechanical measurements and imaging.** (**A**) Microscope stage with the experimental setup installed. The strain gauge sensor and sample holder are positioned within the optical path, aligned with the confocal objective lens. (**B**) Close-up view of the confocal objective lens aligned with the sample mounted on the strain gauge sensor. (**C**) Full view of the experimental setup integrated with the confocal microscope. (**D**) Magnified view of the sample holder under the confocal objective lens. The sample is mounted on the strain gauge sensor, with all wiring routed securely to avoid interference with the optical path. (**E**) Confocal imaging with green laser illumination. The fluorescence signal is captured from the sample mounted on the strain gauge sensor, with clear alignment and minimal interference from mechanical components. (**F**) Close-up of the sample under green fluorescence. (**G**) Confocal imaging with blue laser illumination. (**H**) Magnified view of the sample under blue fluorescence.


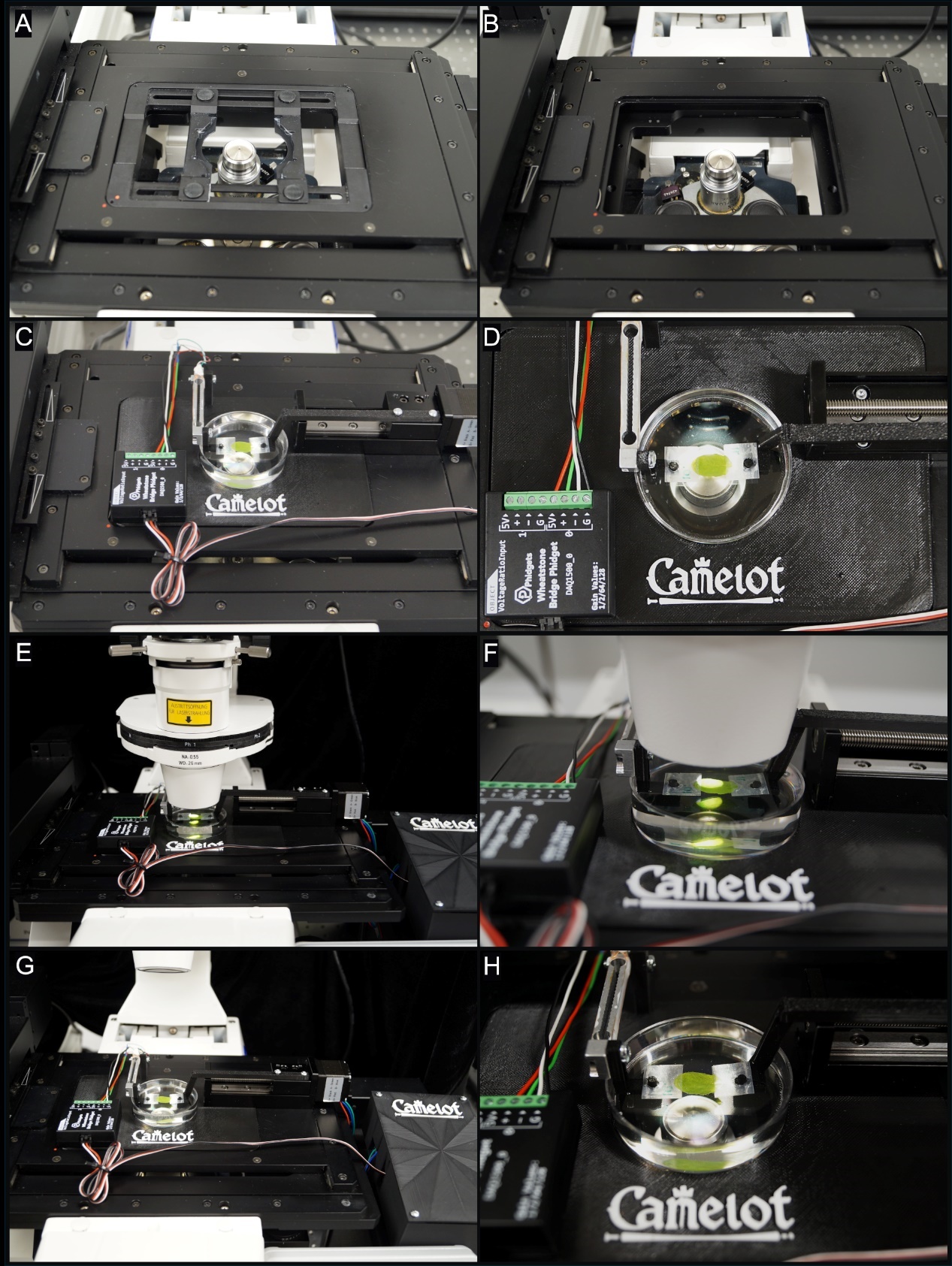


**Additional File 2: Fig. S6: Integration of the experimental setup with an inverted microscope for imaging and measurement.** (**A**) Microscope stage prepared for setup installation. The stage is equipped with adjustable mounting brackets to securely hold the "Camelot" baseplate. (**B**) Microscope stage with the strain gauge mount positioned over the optical path, showing compatibility with the microscope stage design. (**C**) Fully assembled experimental setup installed on the microscope stage. The wiring is neatly routed to avoid interference with the optical path. (**D**) Close-up of the central area of the baseplate. (**E**) The experimental setup under the microscope objective lens. (**F**) Magnified view of the sample under the objective, showing the illuminated sample region. (**G**) Alternative perspective of the setup on the microscope, highlighting the accessibility of all components, including the motor controller and 3D-printed housing for electronics. (**H**) Close-up view of the sample mounted on the strain gauge sensor.


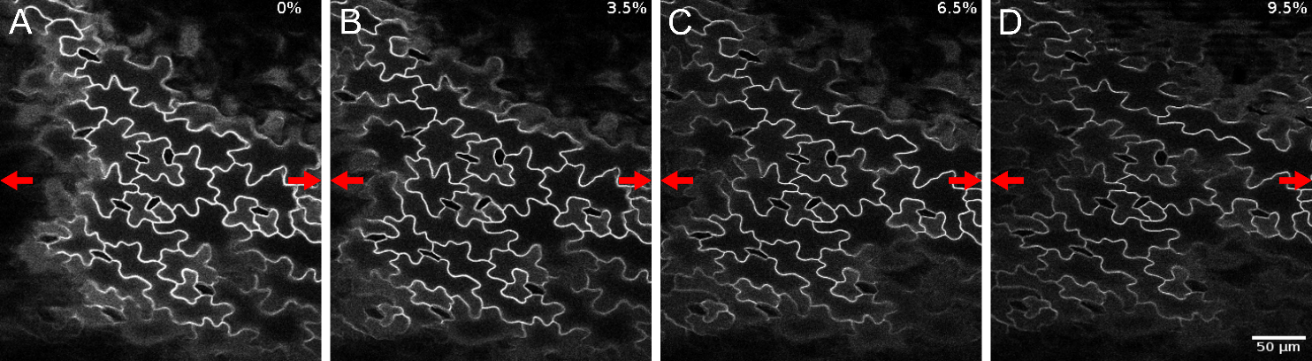


**Additional File 2: Fig. S7:** **Sequence of confocal images during stretching.** A series of confocal images captured during the stretching experiment, showing the progression from 0% strain (**A**) to 3.5% (**B**), 6.5% (**C**), and 9.5% (**D**). Red arrows indicate the direction of stretching. Displacement data are annotated in each frame. Scale bar: 50 µm.
