# Supplementary figures and images for: Camelot: a computer-automated micro-extensometer with low-cost optical tracking
Source: BMC Biol. 2025 Apr 28;23:112. doi: 10.1186/s12915-025-02216-9 (PMC12036183; doi:10.1186/s12915-025-02216-9)

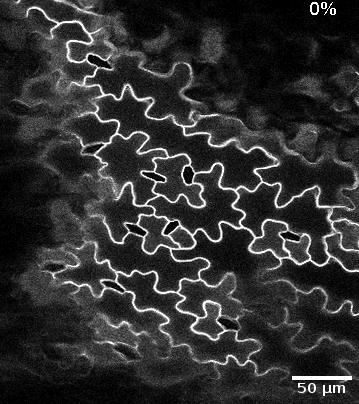

Supplement: Supplementary file 5 — Additional file 5. Movie 2: Time-lapse video of confocal images during stretching. A video composed of sequential confocal images captured during the stretching experiment, showing the progression from 0% to 9.5% strain. Displacement data are annotated throughout the video. Scale bar: 50 µm. [file 12915_2025_2216_MOESM5_ESM.gif]
